# Role of Bicaudal C1 in renal gluconeogenesis and its novel interaction with the CTLH complex

**DOI:** 10.1371/journal.pgen.1007487

**Published:** 2018-07-11

**Authors:** Lucia Carolina Leal-Esteban, Benjamin Rothé, Simon Fortier, Manuela Isenschmid, Daniel B. Constam

**Affiliations:** Ecole Polytechnique Fédérale de Lausanne (EPFL), School of Life Sciences, Swiss Institute for Experimental Cancer Research (ISREC), Lausanne, Switzerland; Stanford University School of Medicine, UNITED STATES

## Abstract

Altered glucose and lipid metabolism fuel cystic growth in polycystic kidneys, but the cause of these perturbations is unclear. Renal cysts also associate with mutations in Bicaudal C1 (Bicc1) or in its self-polymerizing sterile alpha motif (SAM). Here, we found that Bicc1 maintains normoglycemia and the expression of the gluconeogenic enzymes FBP1 and PEPCK in kidneys. A proteomic screen revealed that Bicc1 interacts with the C-Terminal to Lis-Homology domain (CTLH) complex. Since the orthologous Gid complex in *S*. *cerevisae* targets FBP1 and PEPCK for degradation, we mapped the topology among CTLH subunits and found that SAM-mediated binding controls Bicc1 protein levels, whereas Bicc1 inhibited the accumulation of several CTLH subunits. Under the conditions analyzed, Bicc1 increased FBP1 protein levels independently of the CTLH complex. Besides linking Bicc1 to cell metabolism, our findings reveal new layers of complexity in the regulation of renal gluconeogenesis compared to lower eukaryotes.

## Author summary

Polycystic kidney diseases (PKD) are incurable inherited chronic disorders marked by fluid-filled cysts that frequently cause renal failure. A glycolytic metabolism reminiscent of cancerous cells accelerates cystic growth, but the mechanism underlying such metabolic re-wiring is poorly understood. PKD-like cystic kidneys also develop in mice that lack the RNA-binding protein Bicaudal-C (Bicc1), and mutations in a single copy of human BICC1 associate with renal cystic dysplasia. Here, we report that Bicc1 regulates renal gluconeogenesis. A screen for interacting factors revealed that Bicc1 binds the C-Terminal to Lis-Homology domain (CTLH) complex, which in lower eukaryotes mediates degradation of gluconeogenic enzymes. By contrast, Bicc1 and the mammalian CTLH complex regulated each other, and Bicc1 stimulated the accumulation of the rate-limiting gluconeogenic enzyme even in cells depleted of CTLH subunits. Our finding that Bicc1 is required for normoglycemia implies that renal gluconeogenesis may be important to inhibit cyst formation.

## Introduction

Autosomal dominant polycystic kidney disease (ADPKD) is an incurable inherited chronic disorder characterized by progressive kidney enlargement and frequent end-stage renal disease due to numerous fluid-filled cysts that are induced by mutations and loss of heterozygosity in *PKD1* or *PKD2* genes [1]. Complexes of the corresponding transmembrane proteins polycystin-1 (PC1) and polycystin-2 (PC2) are activated at the cell surface by specific WNT ligands to mediate influx of extracellular Ca^2+^, or by mechanical stimulation of primary cilia that induces Ca^2+^ release from intracellular stores in the endoplasmic reticulum [reviewed in 2]. Intracellular Ca^2+^ spikes dampen the levels of cAMP by inhibiting adenylate cyclases V (AC5) and VI (AC6) and by stimulating phosphodiesterase (PDE) activity [2–4]. PC2 and PC1 also negatively regulate AC6 at the level of its mRNA and protein expression [4, 5]. AC6 is important for cystic growth [6] since accumulation of excess cAMP combined with Ca^2+^ restriction stimulates proliferation and cyst enlargement through several pathways, including PKA, EGFR, Src, B-Raf/Erk and mTORC1 signaling [2]. In addition, cystic growth in PC1-deficient kidneys is fueled by a Warburg-like metabolic switch characterized by increased anaerobic glycolysis at the expense of oxidative phosphorylation [7]. How PKD1 attenuates glycolysis is unknown [8].

Large cystic kidneys showing hyperactivation of EGFR, Src and mTORC1 also develop in homozygous mutant *bpk* mutant mice owing to a frame-shifting mutation in one of two alternative transcripts of *Bicc1* [9–12]. Consistent with a role in polycystin signalling, Bicc1 is required to increase PC2 mRNA and protein levels, and its own accumulation is inhibited in *Pkd1* mutant kidneys [13, 14]. Maintaining normal Bicc1 expression is likely important since mutation or loss of a single copy of *BICC1* in human is sufficient to provoke renal cystic dysplasia [15]. Similar to ADPKD kidneys, Bicc1 null mutant kidneys also upregulate AC6 protein levels, accumulate cAMP, and secrete excess Fetuin-A [16, 17]. However, a role of Bicc1 in human ADPKD remains to be defined.

Bicc1 consists of three RNA-binding K homology (KH) and two KH-like domains at the N-terminus, a Gly- and Ser-rich intervening sequence (IVS) and a sterile alpha motif (SAM) at the C-terminus. SAM domains are found in over 4000 proteins (SMART, http://smart.embl-heidelberg.de), often forming dimers or oligomers by head-to-tail self-association [18]. In *bpk* mutant mice, the frame-shifted C-terminus of Bicc1 is abnormally elongated by 149 aberrant amino acids that disrupt SAM-SAM interactions, thus underlining the importance of polymerization for Bicc1 function *in vivo* [19]. Bicc1 and its *Xenopus* homolog xBic-C are thought to inhibit canonical Wnt signal transduction by sequestering cytoplasmic Dishevelled [19, 20] or by directly binding Wnt11 mRNA, respectively [21]. Inhibition of Dishevelled is potentiated by SAM-mediated stabilization of Bicc1 polymers in cytoplasmic foci, whereas KH domains recruit specific mRNAs [16, 19, 20]. Drosophila Bicaudal-C in “Malpighian” tubules (the structures corresponding to renal tubules) has been shown to bind myc mRNA and negatively regulates d-Myc protein levels [22]. Direct Bicc1 targets in kidneys are elusive, but likely include AC6 and protein kinase inhibitor (PKI) α mRNAs to mediate their loading unto miRNA-induced silencing complexes in a process that strictly depends on SAM domain polymerization [16, 19]. However, this is not the only mechanism how Bicc1 regulates mRNA translation. In particular, when bound to, a 3'UTR fragment of Cripto-1 mRNA in early *Xenopus* embryos, xBic-C directly repressed 5' cap-dependent translation, independently of the SAM domain and of miRNA [23]. Furthermore, binding of *Drosophila* Bicaudal-C to its own mRNA has been shown to promote poly(A)-tail deadenylation by the CCR4-NOT complex, or to inhibit it, depending on the developmental context [24]. Consistent with potential roles in translation activation, Bicc1 has also been shown to increase the translation of *Pkd2* mRNA, in this case by protecting the 3'UTR against miR-17 [14]. Furthermore, a novel translation-activating function of Bicc1 mediated by binding to eIF3 on mRNAs specifically at centrosomes seems to require fine-tuning by the orofacial-digital syndrome protein-1 (OFD1) to suppress renal cyst formation [25]. Thus, besides being epistatic to PC1 and PC2, Bicc1 likely mediates multiple functions that could be relevant in PKD and other cystic kidney diseases.

An unbiased approach to identify Bicc1-dependent processes is to analyse its protein interactome. In *Drosophila* ovaries, Bic-C interacts with CCR4-NOT deadenylase complex and with cytoplasmic polyadenylation element binding protein (CPEB) [24, 26]. In addition, *Drosophila* Bic-C co-purified a complex of Me31B, Tral, PABP and Cup that is important to correctly secrete and localize the TGF-α homolog Grk during oogenesis [27]. By comparison, little is known about Bicc1 partners in mammals. In HeLa cells, Bicc1 co-immunoprecipitated with several other KH domain-containing proteins such as Sam68, GLD-1, GRP33, and Qk1 [28]. More recently, Bicc1 was identified as a binding partner of the ankyrin repeat- and SAM domain-containing protein ANKS6, which is mutated in cystic kidneys of a subset of nephronophthisis patients [29, 30]. ANKS6 also binds the related Bicc1-interacting protein ANKS3, suggesting that all three proteins may act in a common cilia signalling pathway [30, 31]. In keeping with a role in cilia signalling, Bicc1 has been detected in primary cilia from proximal tubule epithelial LLC-PK1 cells [32].

Here, we conducted a proteomic screen to identify novel Bicc1-interacting factors. Among 243 proteins that were enriched at least 2-fold by tandem affinity purification (TAP) of Bicc1, we focused on an interaction of Bicc1 with the CTLH complex because the orthologous complex in *S*. *cerevisae* triggers glucose-induced degradation of gluconeogenic enzymes. We show that Bicc1 maintains the levels of the gluconeogenic enzymes fructose-1,6-biphosphatase (FBP1) and phosphoenolpyruvate carboxykinase (PEPCK/PCK1), while inhibiting CTLH complex accumulation in mouse kidneys. Epistasis analysis shows that Bicc1 also stimulates FBP1 protein expression in mIMCD3 and LLC-PK1. Even though the CTLH complex did not inhibit FBP1 in these cell-based models, it negatively regulated Bicc1 protein levels, apparently by competing for the same SAM domain surfaces that are also required for self-polymerization and the silencing of Bicc1 target mRNAs. Our results suggest that Bicc1 is a target of CTLH complex and regulates metabolic processes.

## Results

### A proteomic screen reveals Bicc1 binding to the C-terminal to LisH motif (CTLH) complex

**Fig 1:**
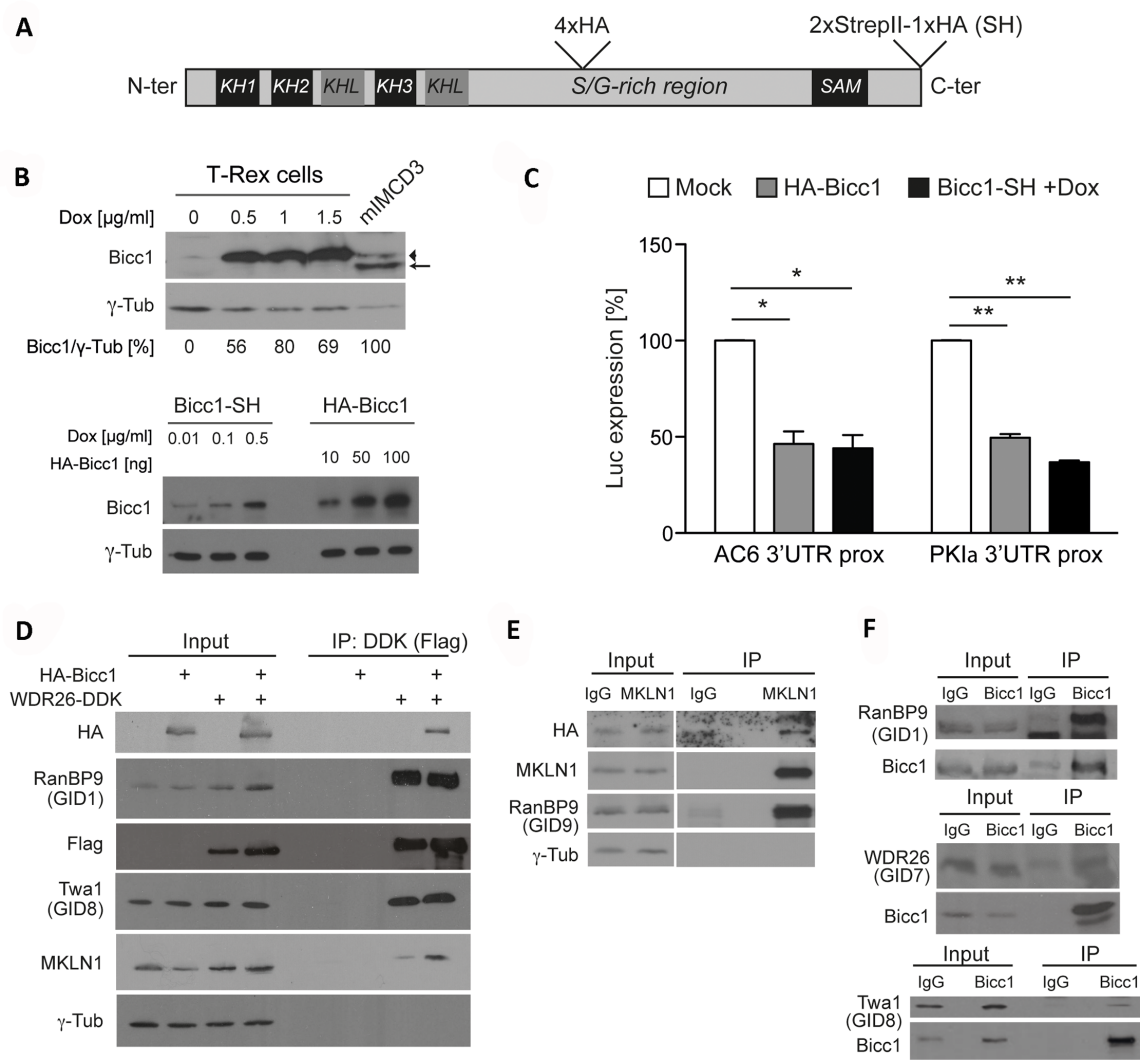
Activity of tagged Bicc1 proteins and coimmunoprecipitation of mammalian CTLH complex subunits. (A) Bicc1-SH tagged with 4 internal and 2 C-terminal StrepII and 1 HA epitopes. Positions of 3 KH (K-Homology domain), 2 KH-like domains (KHL) and one sterile alpha motif (SAM) are indicated. (B) Top panel: Western blot of Bicc1-SH (arrowhead) in Flp-In T-rex cell extracts treated with or without doxycycline for 24 hrs. Traces of Bicc1-SH leaked into the last lane. Densitometric quantification of expression levels relative to endogenous Bicc1 (arrow) in mIMCD3 cells (100%) after normalization to γ-tubulin is shown below. Bottom panel: Western blot of Bicc1-SH induced by the indicated concentrations of doxycycline in comparison to transiently transfected HA-Bicc1. (C) Expression of AC6 and PKIα 3'UTR luciferase reporters in Flp-In T-rex HEK293T cells transfected with HA-Bicc1 or empty vector (mock) or induced with doxycycline to express Bicc1-SH. Luciferase values are relative to co-transfected β-galactosidase. Bars represent mean ± SEM. *p <0.05; **p <0.01. (D) Anti-Flag (DDK) coimmunoprecipitation of HA-Bicc1 and endogenous RanBP9, Twa1 and MKLN1 in HEK293T extracts. Mock transfected cells served as negative control. (E) Anti-MKLN1 coimmunoprecipitation of HA-Bicc1 and endogenous RanBP9 in HEK293T cells. Pre-immune IgG: Negative control. Input and IP samples were on the same gel but shown at different exposure times. (F) Coimmunoprecipitation of RanBP9, WDR26, and Twa1 with endogenous Bicc1 in mIMCD3 cells extract using anti-Bicc1 or pre-immune IgG (negative control).

To identify Bicc1-binding proteins, we purified tandem affinity-tagged Bicc1- StrepII-HA (Bicc1-SH) from a Flp-In T-REx 293 knock-in cell line and analyzed co-purifying proteins by Liquid Chromatography-Mass Spectrometry (LC/MS) (**Fig 1A**). Doxycycline-induced Bicc1-SH expression in HEK293T cells normalized to γ-tubulin reached levels comparable to those of endogenous Bicc1 in mouse inner medullary collecting duct mIMCD3 cells (top panel), but below those of transiently transfected HA-Bicc1 (bottom panel) (**Fig 1B**). To validate that Bicc1-SH was functional, we analyzed its mRNA silencing activity using luciferase mRNA reporters that contain target 3'UTR fragments of AC6 or PKIα [16]. We found that Bicc1-SH and HA-Bicc1 silenced both reporters to a similar extent (**Fig 1C**). LC/MS analysis of Bicc1-SH purified by sequential Strep-tag and anti-HA pull-down identified 243 co-purifying proteins that were enriched at least 2-fold (**S1 Table**). Highly enriched proteins comprised the NOT1 subunit of the CCR4-NOT complex and ANKS3, indicating that our screen detected known Bicc1-interacting factors [24, 31, 33]. Among the novel interactions, we observed 6.5- to 25-fold enrichment of C-terminal to Lissencephaly Homology (CTLH) complex subunits [34], including WD repeat-containing protein WDR26, Ran-binding protein-9 (RanBP9), Macrophage Erythroblast Attacher (MAEA), Two-hybrid-Associated protein With RanBPM 1 (TWA1), Required for Meiotic Nuclear Division 5 homolog A (RMND5a), and 2.3-fold enrichment of Armadillo repeat containing 8 (ARMC8). Only the CTLH subunits Muskelin-1 (MKLN1) and GID4 (formerly C17orf39) were not enriched by Bicc1-SH under these conditions (**Table 1**).

**Table 1:** Mass spectrometric identification of CTLH complex subunits co-purified with TAP-tagged Bicc1-SH.

	CTLH/GID subunit			Unique peptide counts				Fold change^1^
Rank		Gene name	Name	ctrl	IP1	ctrl	IP2	
4	GID7	WDR26	WD repeat-containing protein 26	0	28	0	20	25
17	GID1	RanBP9	Ran-binding protein 9	0	13	0	8	11.5
33	GID9	MAEA1	Macrophage erythroblast attacher	0	11	0	4	8.5
41	GID8	TWA1	Glucose-induced degradation protein 8 homolog	0	7	0	6	7.5
54	GID2	RMND5a	Required for meiotic nuclear division 5 homolog A	0	8	0	3	6.5
200	GID5	ARMC8	Armadillo repeat-containing protein 8	1	5	0	0	2.3

^1^ Fold change was determined after adding +1 to all peptide counts

Several subunits of the mammalian CTLH complex and the orthologous Gid complex in *S*. *cerevisiae* contain Lissencephaly Homology (LisH), CTLH and C-terminal CT11-RanBP9 (CRA) domains, or transducin/WD40 and armadillo repeats [34, 35]. In yeast growing on glucose, the Gid complex consists of 9 subunits required to degrade gluconeogenic enzymes via the proteasomal Pro/N-end rule pathway or via lysosomes [36–38]. The orthologous mammalian CTLH complex has no known function and contained no GID4 under the conditions examined in HEK293 cells [34]. To validate binding to Bicc1, we performed co-immunoprecipitation assays in HEK293T cells that do not express endogenous Bicc1. Anti-Flag immunoprecipitation of DDK-tagged WDR26 co-immunoprecipitated RanBP9, TWA1 and HA-Bicc1 (**Fig 1D**). WDR26 also co-precipitated MKLN1, whereas anti-MKLN1 pulled down HA-Bicc1 together with RanBP9 (**Fig 1E**). Moreover, immunoprecipitation of endogenous Bicc1 in extracts of mIMCD3 cells enriched endogenous RanBP9, WDR26, and Twa1 (**Fig 1F**). These results confirm that mammalian CTLH complexes bind Bicc1.

### Loss of Bicc1 increases WDR26 protein in newborn kidneys

**Fig 2:**
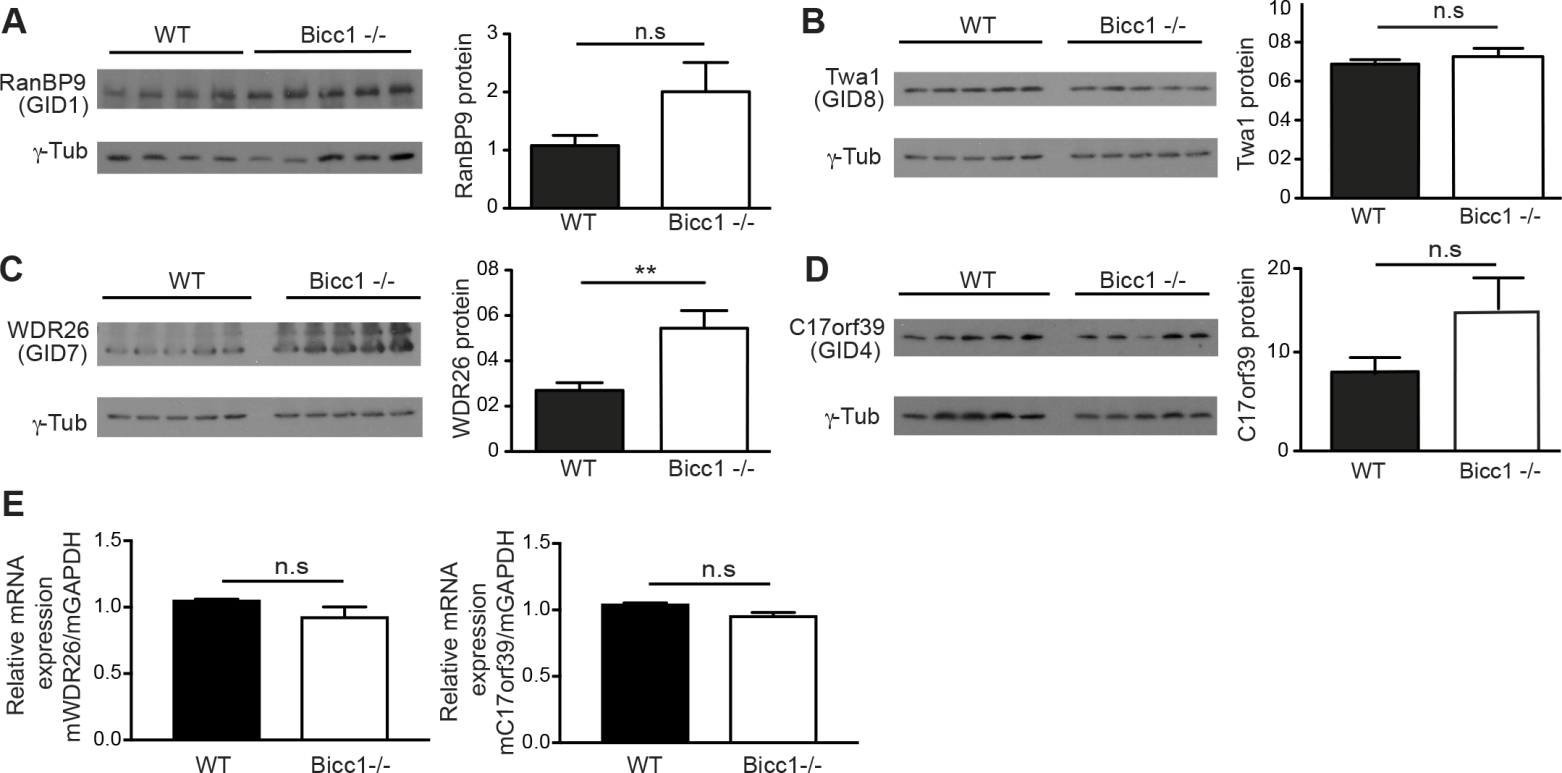
Protein levels of CTLH subunits in Bicc1^-/-^ kidneys. (A-D) Western blot analysis of (A) RanBP9, (B) Twa1, (C) WDR26 and (D) GID4 in newborn WT and Bicc1^-/-^ kidney extracts. Bars represent mean ± SEM. **p <0.01. (E) RT-qPCR analysis of WDR26 and GID4 mRNAs in WT and Bicc1^-/-^ kidneys (n = 3 per genotype). GAPDH mRNA was used for normalization.

To evaluate potential interactions with Bicc1 *in vivo*, we compared the expression levels of CTLH subunits in wild-type (WT) and Bicc1^-/-^ newborn mice. Western blot analysis revealed 2-fold higher WDR26 protein levels in the knockout compared to WT (n = 5, P≤0.01) and a similar trend for GID4 and RanBP9, but not for TWA1 (**Fig 2A–2D**). By contrast, no changes were observed in WDR26 or GID4 mRNAs (**Fig 2E**). These results indicate that Bicc1 attenuates the levels of some but not all CTLH subunits *in vivo*.

### Bicc1 maintains normoglycemia and normal renal expression of the gluconeogenic enzymes FBP1 and PEPCK

**Fig 3:**
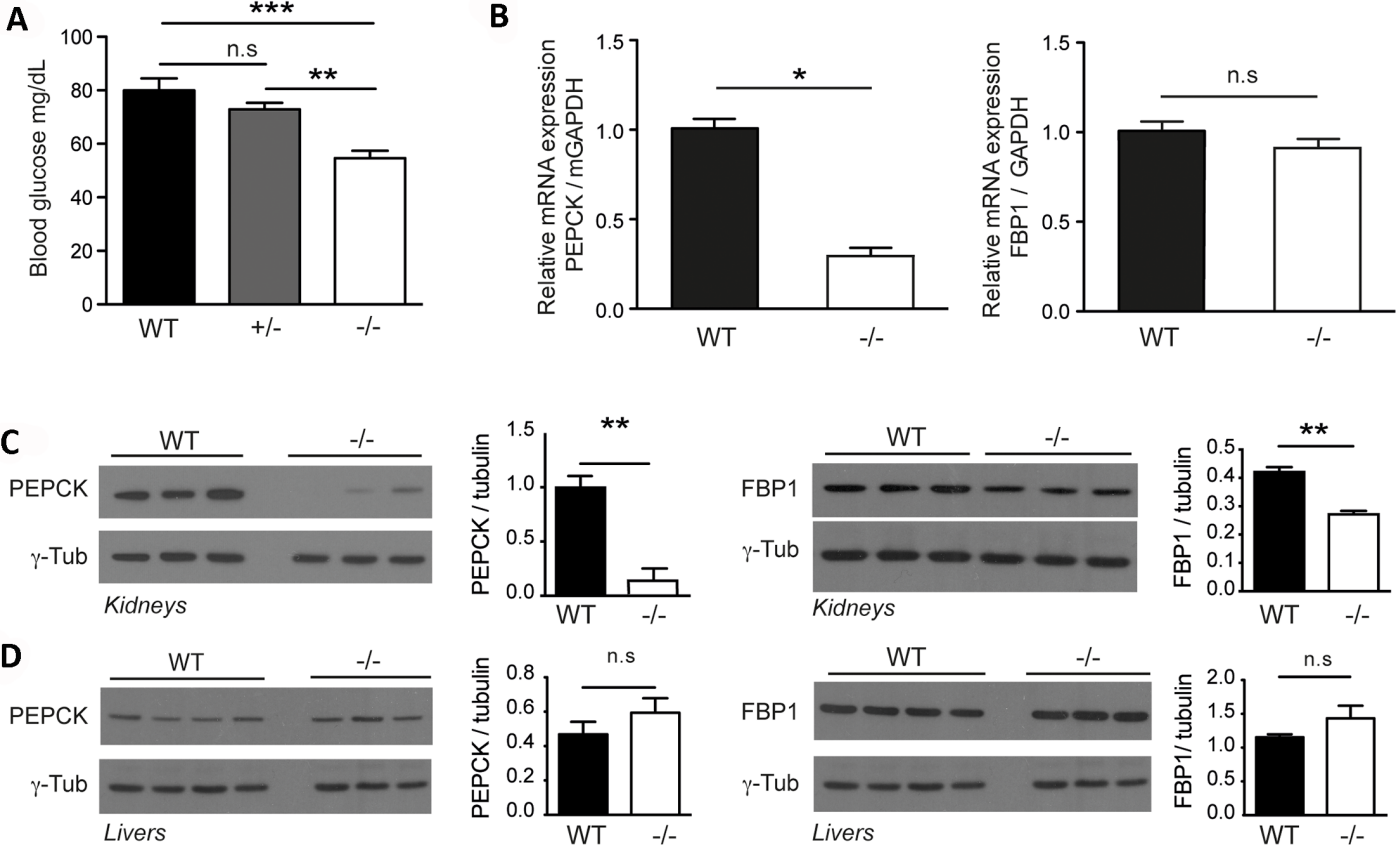
Reduced glycemia and downregulation of FBP1 and PEPCK expression in Bicc1^-/-^ kidneys. (A) Blood glucose measurements in WT and Bicc1^-/-^ mice at postnatal day P2 (n ≥ 6 per genotype). Bars represent mean ± SEM. 1-way ANOVA followed by Bonferroni’s multiple comparisons test **p <0.01; ***p <0.001. (B) RT-qPCR analysis of FBP1 and PEPCK1 mRNA in WT (n = 3) and Bicc1^-/-^ (n = 3) kidneys. GAPDH mRNA was used for normalization. Bars represent mean ± SEM. *p<0.05. (C) Western blot analysis of FBP1 and PEPCK1 in WT (n = 3) and Bicc1^-/-^ kidneys (n = 3) at P2. γ-tubulin was used as loading control. (D) Western blot analysis of FBP1 and PEPCK in WT (n = 3) and Bicc1^-/-^ livers (n = 3) at P2. γ-tubulin was used as loading control.

In *S*. *cerevisiae*, the Gid complex polyubiquitinates FBP1 and PEPCK and induces their degradation to switch from gluconeogenesis to glycolysis in high glucose [37, 39]. To assess glucose metabolism, we first measured blood glucose in WT and Bicc1^-/-^ neonates. Average glucose levels decreased below 60 mg/dl compared to 80 mg/ml in wild-type (**Fig 3A**). Furthermore, RT-qPCR and Western blot analysis revealed 3.4-fold less PEPCK mRNA and 10-fold less protein, whereas FBP1 decreased 2-fold specifically at the protein level and only in Bicc1^-/-^ kidneys (**Fig 3B and 3C**). By contrast in livers, where Bicc1 localizes to cholangiocytes and not to hepatocytes [19], FBP1 and PEPCK expression were unchanged (**Fig 3D**). These results establish that Bicc1 increases PEPCK and FBP1 expression in newborn kidneys and maintains normoglycemia.

### Bicc1 upregulates FBP1 while attenuating the accumulation of several CTLH subunits

**Fig 4:**
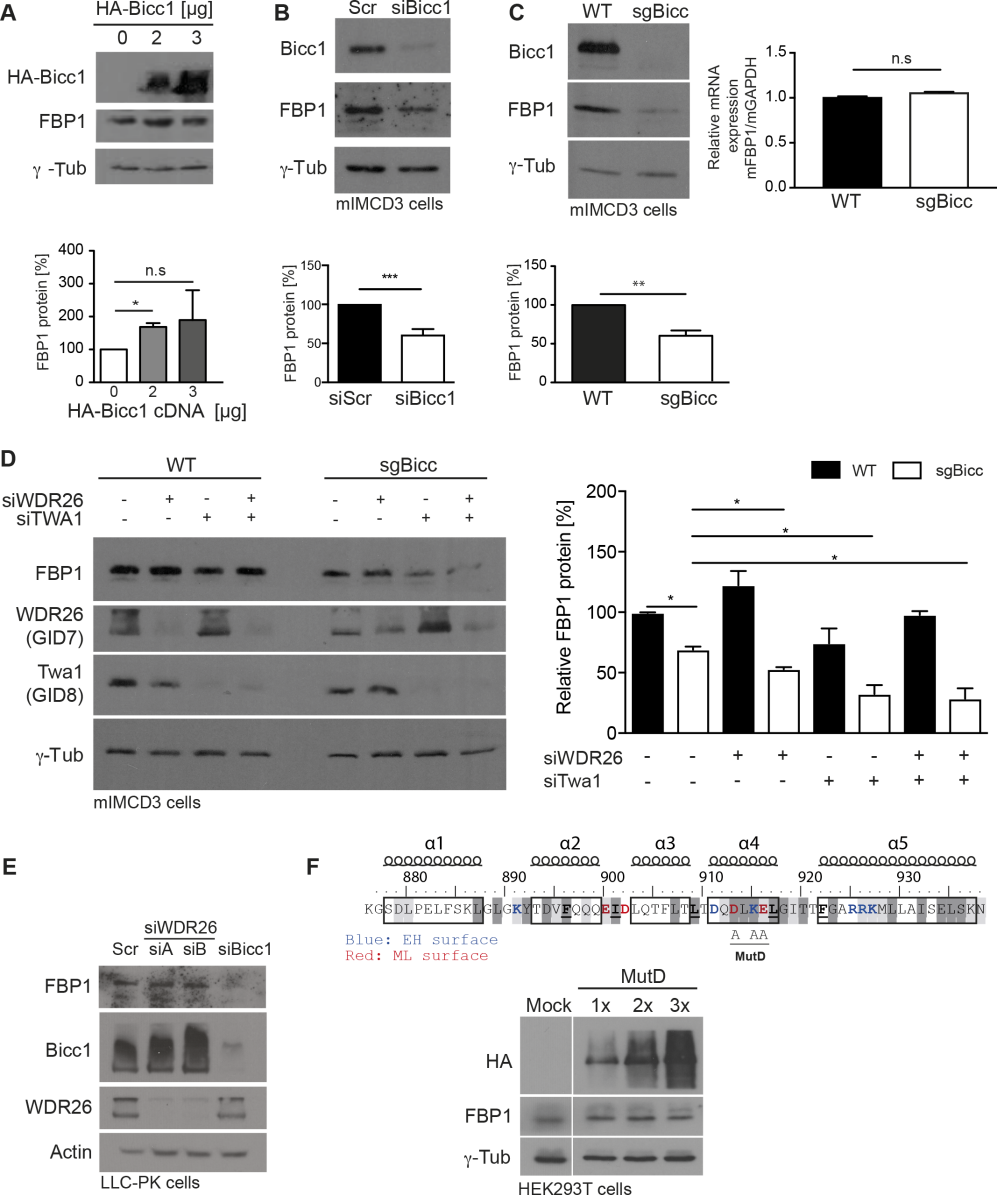
Endogenous Bicc1 in mIMCD3 cells increases FBP1 protein levels independently of its interaction with the CTLH complex. (A) Western blot analysis of FBP1 in HEK293T cells transfected with the indicated dose of HA-Bicc1. Bars represent mean ± SEMs. *p <0.05. (B) Western blot of FBP1 in mIMCD3 48hrs after transfection of scrambled or Bicc1 siRNA. Below: Bars represent mean ± SEMs. ***p <0.001. (C) Western blot and RT-qPCR analysis (right) of FBP1 in mIMCD3 cells (WT) and a representative CRISPR-edited clone (sgBicc1) (n = 3 per genotype). For RT-qPCR GAPDH mRNA was used for normalization. Bars represent mean ± SEMs. **p <0.01 (D) Western blot of FBP1, WDR26 and Twa1 in mIMCD3 transfected with siScr or siWDR26 or siTWA1 siRNAs for 48 hrs. Error bars represent mean ± SEM of two experiments. *p <0.05. (E) Western blots of FBP1, Bicc1 and WDR26 in LLC-PK1 proximal tubule cells transfected with siScr or two different siWDR26 siRNAs (siA, siB) for 48 hrs. Data are representative of two experiments. (F) Western blots of HA-Bicc1 and FBP1 in HEK293T cells transfected with increasing doses of HA-Bicc1 MutD for 24 hrs. Top: Alanine substitutions in Bicc1 MutD (D913;K915;E916/AAA). Charged residues (red and blue) in end helix (EH) and mid loop (ML) surfaces of the dimerization interface in the SAM domain and their conservation determined by ClustalW (grey shading), as well as the positions of 5 α helices are indicated.

Since Bicc1 did not affect *FBP1* transcription, we chose FBP1 to further assess how Bicc1 influences its protein level. Ectopic expression of HA-Bicc1 in HEK293T cells increased the accumulation of FBP1 (**Fig 4A**), whereas RNAi depletion of Bicc1 in mIMCD3 cells [16, 40] decreased it (**Fig 4B**). Alternatively, we inactivated *Bicc1* by CRISPR/Cas9 editing using single guide RNA. While Western blot confirmed the loss of Bicc1 protein, FBP1 levels varied among independent sgBicc clones (**Fig 4C**, **S1 Fig**). To evaluate whether Bicc1 regulates FBP1 through the CTLH complex, we depleted WDR26 or Twa1 or both by RNAi. FBP1 levels did not increase but rather decreased, and only in sgBicc cells, pointing to potentially complex layers of inhibitory mechanisms (**Fig 4D**). Depletion of WDR26 similarly failed to enhance FBP1 accumulation in LLC-PK1 proximal tubule cells, even though RNAi of Bicc1 inhibited it (**Fig 4E**). FBP1 also was not significantly stabilized in cells treated with the proteasome inhibitor MG132 or with H+-ATPase inhibitor Bafilomycin A1, or both (**S1 Fig**). Thus, Bicc1 does not upregulate FBP1 simply by inhibiting the CTLH complex.

To assess the contribution of Bicc1 polymerization, we also tested the regulation of FBP1 by the non-polymerizing D913;K915;E916/AAA mutant Bicc1 MutD [19]. Bicc1 MutD failed to alter FBP1 expression (**Fig 4F**), indicating that the effect of wild-type Bicc1 described above is specific and likely depends on SAM domain-mediated polymerization.

### The CTLH complex and proteasomal degradation attenuate Bicc1 levels

**Fig 5:**
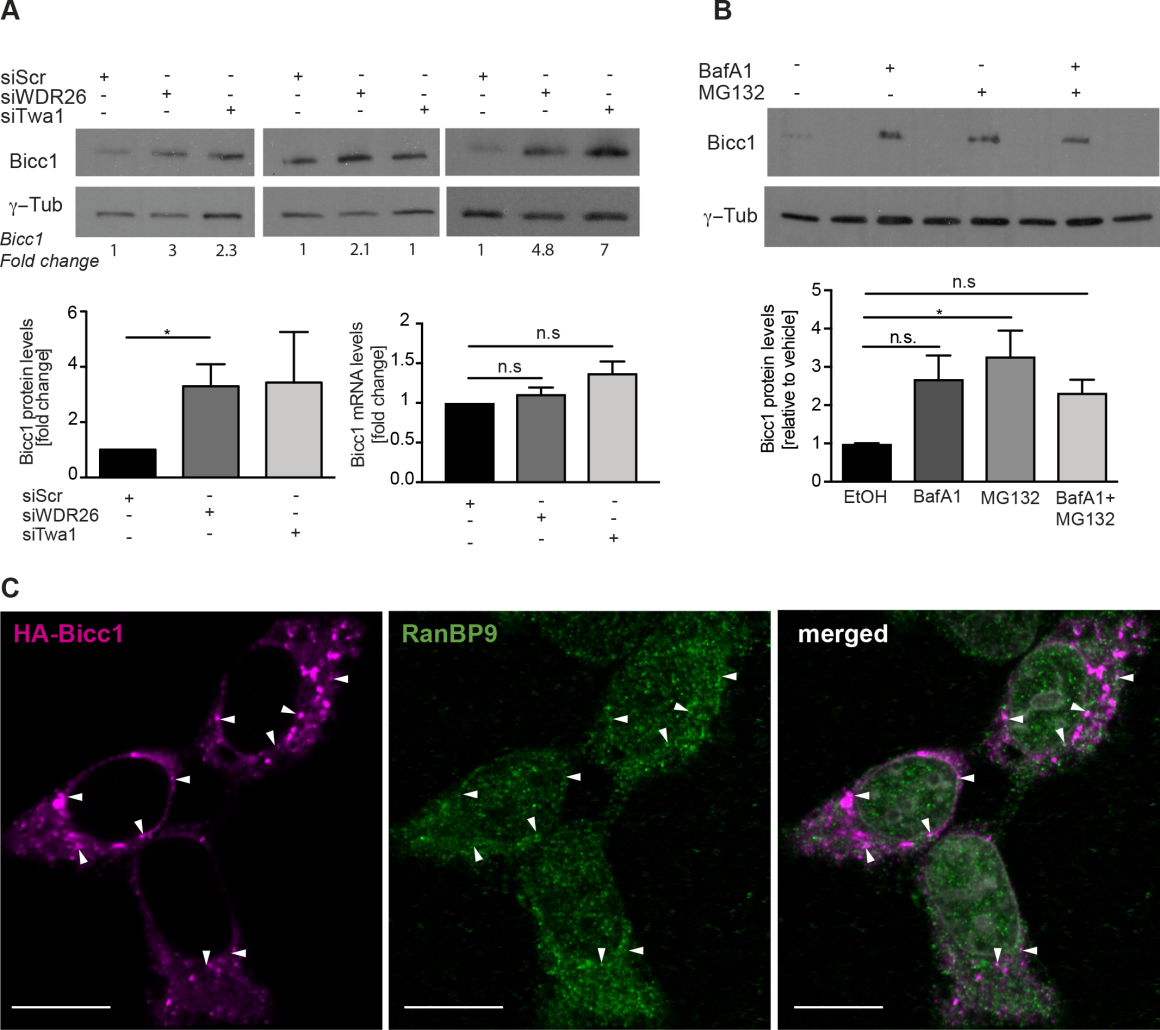
Bicc1 is a target of the CTLH complex. (A) Western blot and RT-qPCR analysis of endogenous Bicc1 in WT mIMCD3 cells treated for 48 hrs with scrambled or WDR26 and Twa1 siRNAs. Quantifications of three experiments show the average fold change in Bicc1 protein (bottom left) and mRNA levels (bottom right) relative to scramble-treated cells in three independent experiments. TATA-box binding protein (TBP) mRNA was used for normalization. (B) Bicc1 protein levels in mIMCD3 cells after treatment with Bafilomycin A1 (BafA1, 100 nM), MG132 [10 μM] or both for 4 hrs. Bars represent mean ± SEM fold changes in Bicc1 protein levels relative to untreated cells in two experiments. *p<0.05. (C) Coimmunostaining of HA-Bicc1 and endogenous RanBP9 in HEK293T imaged at high laser intensities. Bars 20 μm.

Rather than degrading FBP1, CTLH complex may target Bicc1. In keeping with this idea, depletion of CTLH subunits in mIMCD3 cells significantly increased endogenous Bicc1 protein, but not its mRNA (**Fig 5A**). To assess Bicc1 degradation, we treated mIMCD3 cells for 4 hrs with MG132 or Bafilomycin A1 or empty vehicle. Treatment with MG132 increased Bicc1 protein levels without inducing slow migrating intermediates characteristic of polyubiquitination (**Fig 5B**). By contrast, an apparent increase in Bicc1 levels after treatment with the H^+^-ATPase inhibitor Bafilomycin A1 was not statistically significant, and both drugs combined gave variable results, reflecting increased toxicity.

Previously, we have shown that Bicc1 is stabilized in large cytoplasmic foci by self-polymerization of its SAM domain [18, 19]. To distinguish whether the CTLH complex interacts with Bicc1 in cytoplasmic foci or with a non-polymerized pool, or both, we performed co-immunostainings of HA-Bicc1 and endogenous RanBP9 in HEK293T cells. We only observed co-localization with RanBP9 peripheral to and outside bright Bicc1 foci and only at increased laser intensities (**Fig 5C**), indicating that access of CTLH complexes to macroscopically visible Bicc1 self-polymers may be limited.

### The SAM domain mediates Bicc1 binding to the CTLH complex through its polymerization interfaces

**Fig 6:**
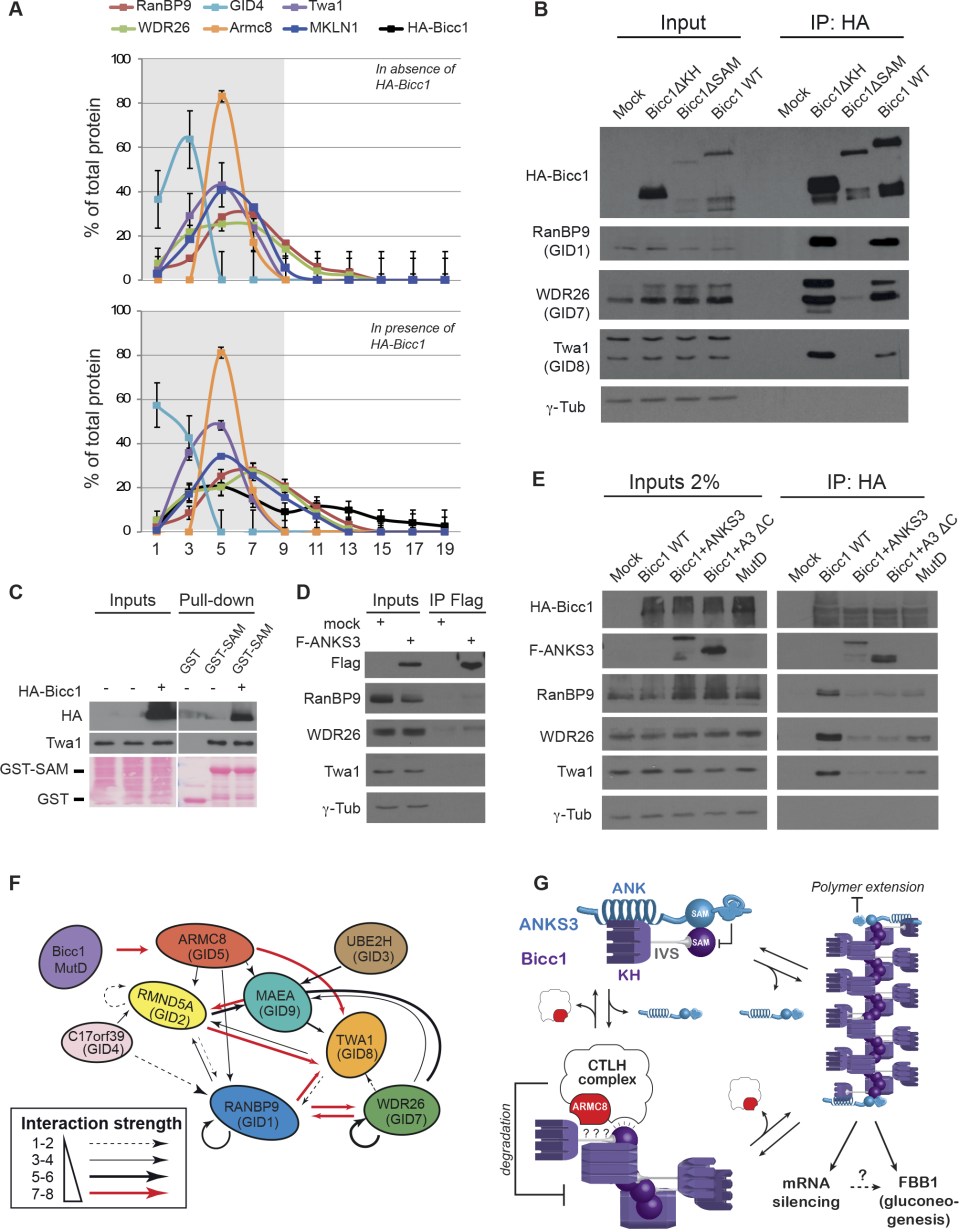
Bicc1 binds the CTLH complex via ARMC8 and dependent on SAM polymer interfaces in competition with ANKS3. (A) Density fractionation of CTLH complex in extracts of empty vector- (upper graph) or HA-Bicc1-transfected HEK293T cells (below). Graphs show percentages of the total of each subunit per fraction. Grey areas denote LMW fractions 1 to 9. Results represent mean ± SEM from 2 experiments. (B) Anti-HA co-immunoprecipitation of CTLH subunits with HA-Bicc1 or its truncated derivatives Bicc1ΔKH and Bicc1ΔSAM in HEK293T cells. Inputs correspond to 2% of the total extract. Mock-transfected cells were used as negative control. (C) Pull down of endogenous Twa1 by a GST fusion protein of Bicc1 SAM domain or GST alone (control) in HEK293T cells transfected with or without HA-Bicc1. Inputs and IPs on the same gel were cropped because of different exposure times. (D) Anti-Flag co-immunoprecipitation assays in HEK293T cell extracts reveal no CTLH complex binding to Flag-tagged ANKS3 above background levels seen with preimmune IgG. (E) Co-immunoprecipitation of CTLH subunits by HA-Bicc1 alone or together with cotransfected Flag-ANKS3 or C-terminally truncated Flag-ANKS3ΔC, or by Bicc1 MutD. Inputs correspond to 2% of the total extract. Inputs and IPs on the same gel were cropped because of different exposure times. (F) Summary of human CTLH complex subunit interactions and binding to Bicc1 MutD mapped by yeast two-hybrid assays. Arrows depict the relative strength of binding in two independent experiments. (G) Recruitment of CTLH complexes via both ARMC8 and polymerization-competent Bicc1 SAM domains accelerates the clearance of LMW Bicc1 oligomers (left), whereas SAM domain polymerization stabilizes Bicc1 in large scaffolds to mediate silencing of specific target mRNAs and to stimulate FBP1 accumulation (right).

To estimate the size of Bicc1-CTLH complexes, we fractionated HA-tagged Bicc1 on sucrose density gradients, using CNOT1 as a control (**Fig 6A**). HA-Bicc1 shifted a peak of CNOT1 from fractions 7–9 to fractions 9–11, all of which also contain ribosomal protein S6 (RPS6) [19] (**S2 Fig)**. By contrast, RanBP9, TWA1, ARMC8, and MKLN1 peaked with low molecular weight (LMW) HA-Bicc1 in fraction 5, and GID4 and WDR26 peaked in fractions 1–3 or 7, respectively. These results indicate that the average size of Bicc1-CTLH complexes is smaller than the ribosome-sized complexes of Bicc1 with CNOT1. Since CTLH complexes did not accumulate in cytoplasmic Bicc1 foci (**Fig 5C**) or in high molecular weight (HMW) fractions (**Fig 6A**), we tested whether they bind Bicc1 independently of its SAM domain. Coimmunoprecipitation experiments in transfected HEK293T cells showed that deletion of the SAM domain inhibited Bicc1 binding to CTLH subunits, whereas deletion of the KH domains did not (**Fig 6B**). To validate that binding involves the SAM domain, we used GST-SAM fusion protein in pull-down assays in HEK293T cell extracts. Irrespective of the presence or absence of exogenous HA-Bicc1, GST-SAM pulled down endogenous TWA1, whereas GST alone did not (**Fig 6C**). As an additional control, we analyzed another SAM domain protein using Flag-tagged ANKS3. Coimmunoprecipitation analysis revealed no Flag-ANKS3 binding to RanBP9, WDR26 or TWA1 above background levels (**Fig 6D**). However, co-transfection of full length ANKS3 or of the truncated mutant ANKS3ΔC lacking the C-terminal region distal of its SAM domain inhibited CTLH complex binding to HA-Bicc1 (**Fig 6E**), indicating that ANKS3 competes with CTLH complex for Bicc1. Previous structure analysis suggests that the SAM domain of ANKS3 interfaces with the ML and EH surfaces of the Bicc1 SAM domain, thereby allowing the bulky C-terminal region of ANKS3 to block Bicc1 polymer elongation [41]. To distinguish whether ML or EH surfaces of the Bicc1 SAM domain or their oligomerization also mediate CTLH complex binding, we mutated them individually [19] (**S3 Fig**). Control mutations MutA and MutF outside the ML and EH surfaces did not inhibit Bicc1 binding to any of the CTLH subunits analyzed, including WDR26, TWA1 and ARMC8. Binding of ARMC8 was also unaffected by mutations MutC or MutE that disrupt the ML or EH surface, respectively. In sharp contrast, disruption of either of these surfaces impaired WDR26 and TWA1 binding, and this defect was not rescued even if Bicc1 MutC and MutE were coexpressed to permit the formation of MutC/MutE dimers (**S3 Fig**). These data suggest that SAM domain oligomerization increases CTLH complex binding.

### Yeast two-hybrid mapping of the mammalian CTLH complex reveals direct binding of ARMC8 to Bicc1 if SAM domain polymerization is inhibited

To further map the topology of CTLH and Bicc1 complexes, we conducted yeast two-hybrid assays. The strongest interactions among CTLH subunits were those of TWA1 with ARMC8, RMND5A, and RANBP9, whereas RanBP9 and RMND5A in turn strongly bound WDR26 and MAEA, respectively, (**Fig 6F, S4 Fig**). GID4 interacted with ARMC8 (GID5), albeit more weakly than its yeast homolog [42]. In contrast, no CTLH subunit alone directly associated with Bicc1, or *vice versa*. To test whether docking sites are buried in Bicc1 polymers, we coexpressed individual CTLH subunits with polymerization mutant Bicc1 MutD. Compared to wild-type, Bicc1 MutD showed increased binding of ARMC8 in two-hybrid assays, but not of other individual CTLH subunits (**Fig 6F, S5 Fig**). To independently validate the influence of SAM domain polymerization, we repeated the TAP-tag purification using Bicc1 MutD, and at lower salt concentrations that allowed Bicc1/CTLH complexes to retain MKLN1 (**Table 2**). We found that compared to WT, Bicc1 MutD co-purified significantly less CTLH complex, even though binding to other proteins such as CAD, FASN and ACACA was not inhibited (**Table 2**).

**Table 2:** Mass spectrometric identification of CTLH complex subunits co-purified with TAP-tagged Bicc1 MutD.

	CTLH/GID subunit			Fold enrichment of peptides ^1^	
Rank		Gene name	Name	Bicc1 WT	Bicc1 MutD
1	GID7	WDR26	WD repeat-containing protein 26	110	31
49	GID9	MAEA1	Macrophage erythroblast attacher	42	5
71	GID8	TWA1	Glucose-induced degradation protein 8 homolog	38	4
166	GID5	ARMc8	Armadillo repeat-containing protein 8	27	2
256	GID1	RanBP9	Ran-binding protein 9	23	6
264	MKLN1	MKLN1	Muskelin	22	4
328	GID2	RMND5a	Required for meiotic nuclear division 5 homolog A	20	1
110	-	ANKS3	Ankyrin repeat and SAM domain-containing protein 3	32	10
26	-	CAD	carbamoyl-phosphate synthetase 2, aspartate transcarbamylase, dihydroorotase	50	46
37	-	FASN	Fatty acid synthase	46	49
1452	-	ACACA	Acetyl-CoA-carboxylase alpha	5	5

^1^ Data represent the total peptide counts from 2 experiments. The fold change was calculated by adding +1 to all counts.

Taken together, these data suggest that the polymerization interfaces of the Bicc1 SAM domain act together with SAM-independent ARMC8 binding to recruit CTLH complexes (**Fig 6G**).

## Discussion

Cystic growth in ADPKD reportedly involves anaerobic glycolysis accompanied by a decrease in the expression of gluconeogenic enzymes [7]. Here, a proteomic screen and validation by coimmunoprecipitation, yeast two-hybrid and density gradient fractionation assays revealed that Bicc1 interacts with the CTLH complex, the mammalian ortholog of the Gid complex that mediates degradation of gluconeogenic enzymes in *S*. *cerevisiae*. CTLH complexes recruited Bicc1 both independently of SAM domain polymerization via ARMC8 and through SAM domain surfaces that can be blocked by ANKS3 or buried in large Bicc1 self-polymers, suggesting CTLH complexes target Bicc1 oligomers devoid of an ANKS3 cap. Our analysis in cultured mIMCD3 cells indicates that CTLH complex curbs the levels of Bicc1 but not of the gluconeogenic enzyme FBP1. Nevertheless, deletion of *Bicc1* in mice led to hypoglycemia and diminished the expression of FBP1 and PEPCK specifically in kidneys but not in liver, correlating with increased accumulation of CTLH complex. These results reveal multi-layered regulation of FBP1 expression by Bicc1 and CTLH complexes: While Bicc1 overall mediates a net increase in FBP1, inhibition of self-polymerizing Bicc1 by the CTLH complex and vice versa provides a mechanism to adjust the levels of Bicc1.

### The protein interactome of Bicc1 suggests a role in metabolism

Bicc1 and the ankyrin and SAM domain proteins ANKS3 and ANKS6 all suppress renal cysts and can coprecipitate each other [29–31]. In our interactome screen, stringent co-purification with TAP-tagged Bicc1 significantly enriched ANKS3 but not ANKS6, confirming that Bicc1 more tightly binds ANKS3 and independently of ANKS6 [43]. Since recent metabolomic analysis links ANKS3 to amino acid metabolism [44], future studies should investigate whether this process also depends on Bicc1. TAP-tag purification of Bicc1 also enriched several CCR4-NOT deadenylase subunits including CNOT1, NOT-5, -6/6L and -9. In addition, we found that Bicc1 can bind to the multifunctional enzyme carbamoyl-phosphate synthetase 2, aspartate transcarbamylase, dihydroorotase (CAD), which is rate-limiting for pyrimidine synthesis [45], and acetyl-CoA carboxylase alpha (ACACA) and fatty acid synthase (FASN) which function sequentially in fatty acid synthesis [46]. While regulation of these enzymes by Bicc1 remains to be tested, these interactions point to potential roles in several metabolic pathways.

### Known functions of CTLH subunits

Here, we focused our attention on the CTLH complex. The core of the homologous Gid complex in *S*. *cerevisiae* is formed by Gid1, Gid5 and Gid9, held together by Gid8. In turn, Gid5 and Gid9 recruit Gid2 [47, 48]. Gid2 and Gid9 are RING domain E3 ubiquitin ligases. Each contains a CTLH motif that is also found in Gid1 and Gid8 and in mammalian RanBP9 (GID1), RMND5a (GID2), TWA1 (GID8), MAEA (GID9), WDR26 (GID7) and in MKLN1 [34, 47]. The CTLH subunit most enriched by TAP tag-purified Bicc1 was WDR26, a cytoplasmic WD40 domain protein that can recruit substrates to the E3 Ub ligase CUL4 and inhibit MAPK-induced activation of serum response transcription factors [49, 50]. Bicc1 also enriched the CUL4-binding protein DDB1, but no CUL4. Possibly, WDR26 and/or DDB1 are substrate-specific adaptors for more than one E3 Ub ligase. RanBP9 was discovered as a partner of Ran, which regulates mitotic spindle assembly and nuclear and ciliary protein import [51–53]. RanBP9 also binds the KH domain protein Fragile Mental Retardation (FMRP) [54]. However, two-hybrid assay detected no RanBP9 interaction with Bicc1 KH domains.

### Bicc1 attenuates renal expression of CTLH complex subunits

Bicc1 significantly diminished the accumulation of the CTLH subunit WDR26, and we noted a similar trend for RanBP9 and GID4. In *S*. *cerevisiae*, the Gid complex is activated by the accumulation of Gid4 within minutes after addition of glucose to degrade gluconeogenic enzymes [37, 38]. A function for human GID4 is elusive. We detected GID4 in HEK293T cells and found that it co-fractionated with other CTLH subunits in sucrose density gradients. GID4 also interacted with RanBP9 and RMND5a in two-hybrid assays. Although this topology differs from *S*. *cerevisiae* where Gid4 is recruited by Gid5 [42], these data show that human GID4 can bind the mammalian CTLH complex. TAP-tagged Bicc1-SH copurified CTLH complexes that contained no detectable GID4. Possibly, Bicc1 reduces GID4 expression or inhibits GID4 recruitment to mammalian CTLH complex. Although, since GID4 expression was low and since binding to CTLH complexes is below detection even in the absence of Bicc1 [34], we could not conclusively test this hypothesis.

### The CTLH complex binds Bicc1 in part via its self-polymerizing SAM domain and limits Bicc1 accumulation

We found that depletion of CTLH complex subunits by RNAi increased endogenous Bicc1 levels in mIMCD3 cells. In the simplest model, the CTLH complex accelerates Bicc1 turnover by direct binding. In keeping with this idea, Bicc1 interacted with the CTLH subunit ARMC8 in two-hybrid assays. ARMC8 has been shown to reduce the accumulation of E-cadherin and of α- and β-catenin to promote cell invasiveness in various cancers [55–57]. ARMC8 targets α-catenin for proteasomal degradation independently of polyubiquitination, despite associating with E3 ligases of the CTLH complex [47, 58]. In two-hybrid assays, ARMC8 bound polymerization mutant but not wild-type Bicc1 or its isolated KH or SAM domains. Furthermore, in mammalian cells, ARMC8 and other CTLH subunits clearly segregated from HMW Bicc1 polymers during density gradient fractionation. SAM domain sequestration in HMW polymers may reduce direct access of ARMC8 to the intervening sequence between KH and SAM domains. In keeping with this model, only a diffusely distributed Bicc1 pool peripheral to microscopically visible cytoplasmic polymers overlapped with RanBP9 foci. Nevertheless, the Bicc1 SAM domain and its polymerization interfaces reinforced binding to assembled CTLH complexes, pointing to cooperativity among its subunits. In keeping with this idea, truncated Bicc1ΔSAM or polymerization mutants only inefficiently coimmunoprecipitated the subunits WDR26 and TWA1, and ARMC8 binding to polymerization mutant Bicc1 MutD during 2-step tandem affinity purifications was weakened compared to wild-type Bicc1. CTLH complex recruitment was also inhibited by overexpressed ANKS3, which competes for free Bicc1 SAM domain interfaces to cap their self-polymerization [43]. Taken together, these observations suggest that ARMC8 primes Bicc1 for recruiting additional CTLH subunits to exposed SAM domains. While the CTLH complex is not the only regulator of Bicc1 degradation, our finding that SAM domain polymerization interfaces mediate this interaction suggests that CTLH complexes preferentially target LMW Bicc1 oligomers at equilibrium with larger polymers when they are uncapped of ANKS3 (**Fig 6G**). It will be interesting to determine whether LMW forms of Bicc1 outside cytoplasmic foci may associate with cilia or centrosomes, and whether their availability is regulated by cell metabolism or *vice versa*.

### Role of Bicc1 in glucose metabolism

A switch of glucose metabolism from glycolysis to gluconeogenesis is important during fasting and at birth when newborns feed on milk, which is low in glucose. During prolonged fasting, up to 25% of gluconeogenesis derives from extrahepatic sources such as kidney and intestine [59]. Prompted by its interaction with the CTLH complex, we tested whether Bicc1 regulates glucose metabolism. Despite hypoinsulinemia, blood glucose levels in Bicc1^-/-^ neonates decreased more than 1.3-fold below wild-type levels. Among possible causes, we considered a role for Bicc1 in gluconeogenesis. Whereas PEPCK converts oxaloacetate into phosphoenolpyruvate, FBP1 catalyzes the rate-limiting step of fructose 1,6-bisphosphate hydrolysis to fructose 6-phosphate. RT-qPCR analysis revealed a 3.4-fold decrease in PEPCK mRNA levels specifically in Bicc1^-/-^ kidneys but not in liver, and both PEPCK and FBP1 were further down-regulated at the protein level. In the absence of glucose, ATP production largely depends on fatty acid oxidation. A resulting increase in the levels of ketone bodies can lead to systemic acidosis if kidneys cannot keep up with acid secretion. Acid secretion into urine requires ammonium production by glutaminolysis, combined with increased gluconeogenesis that drives bicarbonate synthesis and transport into the blood to buffer plasma pH [60]. Normally, hypoglycemia stimulates glucagon-induced upregulation of *PEPCK* expression and gluconeogenesis through cAMP/PKA signalling. Failure to induce renal *PEPCK* expression and gluconeogenesis despite elevated cAMP levels as observed in Bicc1 mutants predicts a defect in acid-base balance, which in turn likely accelerates disease progression. Indeed, accumulation of ammonium in kidney cortex combined with impaired urinary secretion stimulates interstitial nephritis, and it promotes cyst formation in the Han:SPRD rat model of ADPKD [61–63].

### Regulation of FBP1

In *S*. *cerevisiae*, the Gid complex inhibits gluconeogenesis by targeting PEPCK and FBP1 for proteasomal degradation by the proline N-end rule [37, 64]. FBP1 levels decreased almost 2-fold in Bicc1 mutant kidneys without a corresponding decrease at the mRNA level. Depletion of Bicc1 by RNAi also decreased FBP1 protein in cultured mIMCD3 and LLC-PK1 cells, whereas ectopic expression of Bicc1 in HEK293T cells increased it. To test whether endogenous Bicc1 stabilizes FBP1 by inhibiting the CTLH complex, we depleted the subunits TWA1 or WDR26. Knockdown of the CTLH complex by RNAi did not stabilize FBP1. We also observed no accumulation of FBP1 intermediates characteristic of polyubiquitination in cells treated with MG132. In basal-like breast cancer, FBP1 expression can be transcriptionally repressed by Snail1 to stimulate glycolysis and confer a metabolic advantage to cancer stem cells [65], and nearly all renal cell carcinomas deplete FBP1 to promote their aggressiveness [66]. Future studies should investigate whether this process involves Bicc1 or its interaction with CTLH complexes.

## Materials and methods

### Antibodies and Western blot analysis

For Western blot analysis, the following antibodies were used according to manufacturer's instructions: Anti-PEPCK 1:1000 (Abgent), anti-MAEA 1:1000 (R&D systems), anti-C17orf39 1:500 (Aviva), anti-WDR26 1:1000 (Bethyl Lab), anti-Twa1 1:1000 (Proteintech), anti-FBP1 1:500 (Sigma), anti-RanBP9 1:1000 (Abcam), anti-Armc8 1:500 (Santacruz), anti-Bicc1 1:1000 (raised by Proteogenix), anti-HA 1:1000 (Sigma), anti-γ-Tubulin 1:1000 (Sigma), anti-Actin clone C4 1:1000 (Merck). We used densitometric analysis for protein band quantification.

### Analysis of kidneys and plasma glucose levels in Bicc1 mutant mice

Bicc1^+/-^ heterozygous mice on a C57Bl/6 mouse genetic background were maintained in ventilated cages at the animal facility of the Ecole Polytechnique Fédérale de Lausanne (EPFL) [20]. All animal experiments were approved by the Veterinary Service of the Swiss canton of Vaud or by the Institutional Animal Care and Use Committee and adhered to the guidelines in the Guide for the Care and Use of Laboratory Animals (National Research Council. 2011. Guide for the care and use of laboratory animals, 8th ed. National Academies Press, Washington, DC). Newborn mice were decapitated at postnatal day P2, and total blood glucose was measured using the Accu-Check Aviva Nano glucometer (Roche). Kidney and livers were immediately snap frozen in liquid nitrogen after dissection. For use, the tissues were grinded with a pestle in lysis buffer and sonicated using a Bioruptor device (Diagenode). After centrifugation, protein concentration was quantified using Bradford assay (Bio-Rad).

### Plasmids

To generate TAP-tagged MmBicc1 and MmBicc1-D913K915E916/AAA (Bicc1 MutD), a previously described HA-tagged mouse Bicc1 cDNA [20] and HA-tagged mouse Bicc1 MutD [19] were amplified by PCR and fused in-frame as a Kpn I restriction fragment to the SH tag in the inducible expression vector pcDNA5/FRT/TO (courtesy of Aebersold Lab). Alanine substitutions of residues D913, K915 and E916 in HA-Bicc1 have been described previously [19]. Briefly, the mutated DNA fragment was amplified by overlap extension PCR and then subcloned between Bgl II and Xba I sites of pCMV-SPORT6::HA-Bicc1. DNA fragments for Y2H assays were cloned by PCR in plasmid pACT2 distal to the GAL4 transactivation domain coding sequence, or in plasmid pGBKT7 distal to the GAL4 DNA-binding domain coding sequence using primers with unique restriction sites. The luciferase reporter plasmids Luc-AC6 3’UTRprox and Luc-PKIα 3’UTRprox in the vector pCS2+ have been described [16].

### Cell culture and transfections

HEK293T and mIMCD3 cells were cultured in DMEM medium (Sigma) supplemented with 10% fetal bovine serum (FBS; Sigma), glutamine (1%; Invitrogen), and gentamicin (1%; Invitrogen). LLC-PK1 cells were cultured in DMEM with 5.5 mM D-glucose, supplemented with 10% fetal bovine serum, 100U/ml penicillin, 100ug/ml streptomycin. These and all other cell lines in this study were negative for mycoplasma as determined by an ELISA-based assay (Roche). Expression vectors were transfected using jetPEI transfection reagent (Polyplus) according to the manufacturer's instructions. Small interfering RNAs against MmWDR26 (TAAAGGCTTTAGCTCATTCAGGTCA), MmTwa1 (CCGACTCATCATGAACTAC) and MmBicc1 (CCAACCACGUAUCCUAUAATT) or SsWDR26 A (CCTCATGCAAGAGTCAGGATGTCGT), SsWDR26 B (AATAGGACAGCACTTGAATGG) or scrambled control (Microsynth) were transfected during 48 hrs using INTERFERin transfection reagent (Polyplus). CRISPR/Cas9 editing of Bicc1 mIMCD3 cells was carried out using the guide sequences 5’-GCGAGCGCAGCACCGACTCGCCGG-3’ were cloned into the expression vector PX458 containing GFP-tagged Cas9 [67]. The resulting sgRNA/Cas9 expression vector were transfected and after 24h, the cells were trypsinized, washed with PBS and resuspended in PBS/1% FBS for single cell sorting for GFP by FACS into 96-well plate containing complete medium.

### Indirect immunofluorescence

HEK293T cells were transfected with 1 μg of HA-Bicc1 in 6 well plates. After 24 hrs, the cells were plated on coverslips. At 48 hrs post-transfection, cells were washed with phosphate-buffered saline (PBS) and methanol-fixed during 10 mins at -20°C. Cells were washed again with PBS and incubated with blocking solution containing 1% BSA for 1 hr at room temperature. Mouse anti-HA and rabbit anti-RanBP9 antibodies were diluted 1:500 and 1:100, respectively, in blocking solution and added to the cells during 2 hrs at room temperature. After washing with PBS, cells were incubated with Alexa 647-conjugated secondary donkey anti-mouse and Alexa 488-conjugated donkey anti-rabbit IgG during 1 hr at room temperature in the presence of 4’,6-diamidino-2-phenylindole (DAPI). Pictures were acquired by confocal microscopy using a Zeiss LSM700 microscope.

### TAP-tag purification

To generate the inducible MmBicc1-SH stable cell line, we co-transfected Flp-InT-REx HEK293 cells (Invitrogen) with pOG44 Flp recombinase expression plasmid (Invitrogen) and with pcDNA5/FRT/TO vector containing MmBicc1-SH. Correct integration by Flp-mediated homologous recombination gave rise to hygromycin-resistant clones that were isolated and validated for MmBicc1-SH expression after 24 hrs of doxycycline induction. For tandem affinity purification [68], 3 dishes of Bicc1-SH T-Rex cells were treated without (control) or with doxycycline (1 μg/ml) during 24 hours. Cells were rinsed and scraped from dishes in Tris-buffered saline (TBS) containing 150 mM NaCl in 50 mM Tris^.^HCl pH 7.4, followed by extraction with TBS containing 1 mM DTT, 0.05% NP-40, phosphatase inhibitor cocktail 3 (Sigma), RNAse inhibitors (Promega) and protease inhibitor cocktail (Roche). After sonication and centrifugation at 10'000 × g for 10 min, supernatants were incubated on a rotating wheel for 2 hrs at 4°C with Strep-Tactin Sepharose resin (IBA). After incubation, resins were loaded in Mobicol columns (MoBiTec) and washed with 10 ml 0.05% NP-40 in TBS, and eluted on ice using desthiobiotin 3X (containing 400 mM NaCl) during 15 min as described [69]. For the second purification, eluates were incubated with anti-HA agarose beads (Sigma, A2095) during 2 hrs at 4°C. After incubation, beads were loaded on Mobicol columns, rinsed with 10 ml Tris^.^HCl pH 7.4 (10 mM) containing 100 mM NaCl, 2.5 mM MgCl_2_, 1 mM DTT, and 0.02% NP-40, and eluted using 125 mM HCl into a vial containing 50 μl of Triethylammonium bicarbonate (TAEB) neutralization buffer (Sigma). Eluates were concentrated in 40 μl using Amicon Ultra 0.5 ml 3K (Millipore). TAP-tag purification of Bicc1-MutD-SH was done with buffer containing Tris^.^HCl pH 7.6 (30 mM), 150 mM NaCl, 1 mM MgCl_2,_ 0.05% NP-40, phosphatase inhibitor cocktail 3 (Sigma), RNAse inhibitors (Promega) and protease inhibitor cocktail (Roche). Strep elution was done using desthiobiotin 2X (containing 300mM). Elution from HA bead was done as described above. Laemmli buffer was added to the eluates and boiled samples were loaded in a 4–15% Mini-PROTEAN TGX precast Gel (BioRad).

### LC-MS/MS analysis

LC-MS/MS analysis was done at the Proteomic core facility (PCF) at Ecole Polytechnique Fédérale de Lausanne (EPFL). Peptides were extracted from Coomassie Brilliant blue R-250 stained gel slices and subjected to tryptic digestion. Reverse phase separations were performed on a Dionex Ultimate 3000 RSLC nano-UPLC system (Thermo Fisher Scientific) connected online an Orbitrap Elite Mass Spectrometer (Thermo Fisher Scientific) piloted with Xcalibur (version 2.1) and Tune (version 2.5.5), as described in [70]. Samples were analyzed using Mascot (version 2.6, Matrix Science, Boston, MA, USA) set up to search the human subset of the UniProt database (Release 2017_02). For visualization and validation, MS/MS data was entered in Scaffold 4 (Proteome Software Inc., Portland, OR). The peptide identification threshold, and the protein identification threshold based on matches with at least 2 identified peptides were set at 1% FDR. The MS analysis was conducted in duplicates from two biological replicates. For the semi-quantitative analysis of the MS data, a fold enrichment has been calculated by comparison with the background found in the control condition. The values have then been normalized over the fold enrichment obtained for the bait protein. The presented values correspond to the average of the normalized fold enrichments obtained in the two replicates.

### Luciferase reporter assays

To measure luciferase expression, HEK293T cells were plated in 24-well plates and transfected on the following day with the indicated plasmids (100 ng/well) and with a lacZ expression vector (50 ng/well) in triplicate samples using jetPEI (Polyplus Transfection). 24 hrs after transfection, cells were lysed in buffer containing 25 mM Tris-phosphate, pH 7.8, 2 mM DTT, 2 mM 1,2-diaminocyclohexanetetraacetic acid (CDTA), 10% glycerol, and 0.5% Triton X-100. Cell extracts were diluted 20-fold, and luminescent counts detected in a Centro XS3 LB960 microplate luminometer (Berthold Technologies). Values were normalized to β-galactosidase activity measured by spectrophotometry in a Safire2 microplate reader. Results represent mean values from at least three independent experiments. Error bars show standard errors of the mean (SEM). Student's t-test was used to calculate P values.

### Co-immunoprecipitation

HEK293T cells were transfected with the indicated expression plasmids in 10 cm dishes (we used 2 μg/dish). 24 hrs after transfection, cells were washed with PBS and proteins were extracted with lysis buffer containing 1X TBS, 1mM DTT, 0.05% NP-40, 1x protease inhibitor cocktail (Roche) and phosphatase inhibitor cocktail 3 (Sigma). Endogenous Bicc1 was immunoprecipitated from mIMCD3 cell extracts. In brief, confluent cells in 10 cm dishes were washed with PBS and resuspended in extraction buffer as described above. After sonication and centrifugation at 10'000 × g for 10 min, supernatants were incubated on a rotating wheel for 2 hrs at 4°C with anti-HA agarose antibody or anti-FLAG M2 affinity gel (Sigma), or with protein G Sepharose beads (GE Healthcare) precoated with Bicc1 custom antibody [19] or pre-immune IgG (R&D Systems). Beads loaded on Mobicol columns were washed with 10 ml of Tris^.^HCl pH 7.4, 100 mM NaCl, 2.5 mM MgCl_2_, 1 mM DTT, and 0.02% NP-40 washing buffer and resuspended in Laemmli buffer. Elutions were fractionated on SDS-PAGE gels and analyzed by Western blotting.

### GST pull-down

Recombinant GST-Bicc1 SAM domain was produced in the E. coli BL21 strain (Novagen) as previously described using pGEX-1λT expression vector [16]. GST fusion protein were purified from cell extract under native conditions, using Glutathione Sepharose 4B (GE Healthcare) in 50 mM Tris.HCl pH 8, 200 mM NaCl, 1 mM DTT according to manufacturer's instructions, rinsed twice with 20 mM Tris.HCl pH 7.4; 750 mM NaCl; 1 mM DTT washing buffer followed by Tris^.^HCl pH 7.4, 20 mM; NaCl 200 mM; DTT 1 mM washing buffer and eluted by addition of 20 mM glutathion. Confluent HEK293T cells in 10 cm dishes were lysed in TBS buffer containing 1 mM DTT, 0.05% NP-40, Phosphatase inhibitor cocktail 3 (Sigma), RNAse inhibitors (Promega) and Protease inhibitors cocktail (Roche). After sonication and centrifugation at 10'000 × g for 10 min, supernatants were incubated on a rotating wheel for 2 hrs at 4°C with glutathione-Sepharose 4B beads saturated with GST-Bicc1 SAM or GST alone (negative control). After washing on Mobicol columns with 5 ml of 20 mM Tris^.^HCl pH 7.4 containing 200 mM NaCl, 2 mM MgCl_2_, 1 mM DTT, and 0.05% NP-40, the beads were resuspended in Laemmli buffer. Eluates were fractionated on SDS-PAGE gels and analyzed by Western blotting. Loading of GST fusion proteins was validated indirectly by Ponceau staining of proteins retained in the gel.

### Sucrose density gradient fractionation

Subconfluent HEK293T cells were transfected with HA-Bicc1 or empty vector in 10 cm dishes (2 μg DNA/dish) using 3 plates per condition. Cell extracts were prepared after 24 hrs as described above for the GST pull-down assay. Continuous 15 to 60% sucrose gradients were prepared as described previously [19]. Fractions were recovered manually starting from the top, fractionated on SDS-PAGE gels, and analyzed by Western blotting. γ-tubulin was used as a control.

### Reverse transcription qPCR

Total RNA from mIMCD3 cells and kidneys was isolated using TRIzol (Life Technologies) and RNeasy plus mini kit (Qiagen) following manufacturer's instructions. Total RNA was digested with RQ1 DNase (Promega). Reverse transcription of cDNA was carried out using SuperScript III reverse transcriptase and Oligo dT (Life Technologies) according to the manufacturer's recommendations. The qPCR was performed in a QuantStudio 6 Flex real-time PCR systems (Applied Biosystems) using goTaq qPCR 2x Master Mix (Promega). Samples were analyzed as triplicates, and expression levels were calculated with the manufacturer’s software using the ΔΔCt method. The PCR primers are described in S2 Table.

### Yeast two-hybrid assay

To assess binding of each CTLH complex subunit to WT Bicc1 or Bicc1- D913K915E916/AAA (MutD), they were each fused to the DNA-binding domain of the GAL4 transcription factor (GAL4‐BD) in the pGBKT7 plasmid (Clontech) as bait proteins. In parallel, we fused them each to the activation domain of the GAL4 transcription factor (GAL4‐AD) in the pACTII plasmid (Clontech) as prey proteins. The reporter gene used in this study is the HIS3 gene required for histidine biosynthesis. To monitor bait and prey interactions, appropriate pACTII (LEU2) and pGBKT7 (TRP1) plasmids were transformed into haploid cells from strain CG1945 (mat a; ura3‐52, his3‐200, ade2‐101, lys2‐801, trp1‐901, leu2‐3, 112, gal4‐542, gal80‐538, cyhr2, LYS2::GAL1UAS‐GAL1TATA‐HIS3, URA3::GAL417‐mers(x3)‐CYC1TATALacZ) and strain Y187 (mat α; gal4, gal80, ade2‐101, his3‐200, leu2‐3,112, lys2‐801, trp1‐901, ura3‐52, URA3::Gal1UAS GAL1TATA‐LacZ), respectively, using the lithium acetate method [71]. After crossing on YPD medium, diploid cells were selected on media suitable for double selection (Leu‐, Trp‐) and then plated on media suitable for triple selection (Leu‐, Trp‐, His‐). Where indicated, 3‐Amino‐1, 2, 4‐triazol (3‐AT) was added as a competitive inhibitor of histidine synthesis to evaluate the strength of the interactions. Growth was assessed after three days of incubation at 30°C. The interactions were confirmed in two independent experiments.

### Statistical analysis

Error bars represent the standard error of the mean (SEM). Two-tail student-t test was used to compare the differences between 2 conditions to calculate p-values. 1-way ANOVA and Turkey’s multiple comparison test was used to compare groups of unpaired values and determine the significance (p-value) of every mean compared to every other mean.

## Supporting information

S1 Fig CRISPR/Cas9-editing of Bicc1 in mIMCD3 cells. (A) Schematic of Bicc1 targeting. (B) Bicc1 and FBP1 Western blot of CRISPR/Cas9 treated mIMCD3 cell clones. (C) FBP1 protein levels in mIMCD3 cells after treatment with Bafilomycin A1 (BafA1, 100 nM), MG132 [10 μM] or both for 4 hrs. Bars represent mean ± SEM fold changes in Bicc1 protein levels relative to untreated cells in two experiments (**p <0.01).
(TIF)

S2 Fig Western blot analysis of density-fractionated HEK293T cell extracts. Extracts of HEK293T transfected with HA-Bicc1 or empty vector (mock) were fractionated on a continuous 15 to 60% sucrose gradient. Arrows indicate the order for collecting the fractions (top to bottom). Inputs and fractionated samples were on the same gel but shown at different exposure times.
(TIF)

S3 Fig Specific mutations in Bicc1 self-polymerization interfaces reduce CTLH complex binding. (A) Positions of mutations in the Bicc1 SAM domain, and their effects on the integrity of EH or ML surfaces [19]. (B) Space filling model of a Bicc1 SAM domain dimer from an angle viewing the positions of the control mutations MutA and MutF in the first or fifth α-helix, respectively, outside the ML and EH surfaces of the SAM-SAM interface. (C) Backside view of a SAM domain heterodimer of co-expressed Bicc1 MutC and Bicc1 MutE associating through their wild-type EH and ML surfaces, respectively, so that MutC or MutE mutations at the extremities prevent polymer extension. (D) As in (C), but with individual SAM subunits rotated along their vertical axis to display frontal views of their EH (left) or ML surface (right). The position of the mutation MutD (purple) encompasses both surfaces. (E) Western blot analysis of the indicated CTLH complex subunits after co-immunoprecipitation with HA-Bicc1 or polymerization mutant derivatives. γ-tubulin was a loading control. Inputs represent 2% of cell extracts. Numbers below each panel indicate the ratio of protein that coprecipitated with the indicated polymerization mutant HA-Bicc1, divided by the amount pulled down by wild-type control. HA-Bicc1 was imaged by a LI-COR Odyssey CLx system to avoid signal saturation. (F) Mean values ± SEM from 4 independent experiments are shown below. P values were estimated using 2-way Anova and Dunnet's multiple comparison test (*p<0.05, **p <0.01, ***p <0.001).
(TIF)

S4 Fig Binding of CTLH subunits to each other in yeast two-hybrid assays. Pairs of CTLH subunits fused to Gal4-AD bait or Gal4-DBD prey proteins induce cell growth if they bind each other. Each CTLH subunit was tested both as bait and prey. Empty Gal4-AD was a negative control. Titration of the competitive HIS3 antagonist 3‐Amino‐1,2,4‐triazol (3AT) served to assess the strength of each interaction. Data are representative of 2 experiments with similar results.
(TIF)

S5 Fig Binding of CTLH subunits to wild-type or Bicc1 MutD. Yeast two-hybrid assays of the indicated bait and prey fusion proteins. Data are representative of 2 experiments with similar results.
(TIF)

S1 Table Proteins enriched by co-purification with TAP-tagged Bicc1 from T-Rex cells. (XLSX)

S2 Table Primers used for RT-qPCR in this study. (TIF)

## References

[1] Ong AC, Harris PC. A polycystin-centric view of cyst formation and disease: the polycystins revisited. Kidney Int. 2015;88(4):699–710. Epub 2015/07/23. 10.1038/ki.2015.207 ; PubMed Central PMCID: PMCPMC4589452. 26200945 PMC4589452

[2] Mekahli D, Parys JB, Bultynck G, Missiaen L, De Smedt H. Polycystins and cellular Ca(2+) signaling. Cellular and Molecular Life Sciences. 2012 Epub 2012/10/19. 10.1007/s00018-012-1188-x . 23076254 PMC3708286

[3] Sharma RK, Das SB, Lakshmikuttyamma A, Selvakumar P, Shrivastav A. Regulation of calmodulin-stimulated cyclic nucleotide phosphodiesterase (PDE1): review. International journal of molecular medicine. 2006;18(1):95–105. . 16786160

[4] Choi YH, Suzuki A, Hajarnis S, Ma Z, Chapin HC, Caplan MJ, et al Polycystin-2 and phosphodiesterase 4C are components of a ciliary A-kinase anchoring protein complex that is disrupted in cystic kidney diseases. Proceedings of the National Academy of Sciences of the United States of America. 2011;108(26):10679–84. Epub 2011/06/15. doi: 1016214108 [pii] 10.1073/pnas.1016214108 . 21670265 PMC3127890

[5] Yanda MK, Liu Q, Cebotaru L. AN INHIBITOR OF HISTONE DEACETYLASE 6 ACTIVITY, ACY-1215, REDUCES cAMP and CYST GROWTH IN POLYCYSTIC KIDNEY DISEASE. American journal of physiology Renal physiology. 2017:ajprenal 00186 2017. 10.1152/ajprenal.00186.2017 . 28747357 PMC5668593

[6] Rees S, Kittikulsuth W, Roos K, Strait KA, Van Hoek A, Kohan DE. Adenylyl Cyclase 6 Deficiency Ameliorates Polycystic Kidney Disease. J Am Soc Nephrol. 2013;25(2):232–7. Epub 2013/10/26. 10.1681/ASN.2013010077 . 24158982 PMC3904559

[7] Rowe I, Chiaravalli M, Mannella V, Ulisse V, Quilici G, Pema M, et al Defective glucose metabolism in polycystic kidney disease identifies a new therapeutic strategy. Nature Medicine. 2013;19(4):488–93. 10.1038/nm.3092 23524344 PMC4944011

[8] Magistroni R, Boletta A. Defective glycolysis and the use of 2-deoxy-D-glucose in polycystic kidney disease: from animal models to humans. Journal of nephrology. 2017 10.1007/s40620-017-0395-9 . 28390001

[9] Cogswell C, Price SJ, Hou X, Guay-Woodford LM, Flaherty L, Bryda EC. Positional cloning of jcpk/bpk locus of the mouse. Mammalian genome: official journal of the International Mammalian Genome Society. 2003;14(4):242–9. 10.1007/s00335-002-2241-0 . 12682776

[10] Dell KM, Nemo R, Sweeney WE Jr., Levin JI, Frost P, Avner ED. A novel inhibitor of tumor necrosis factor-alpha converting enzyme ameliorates polycystic kidney disease. Kidney international. 2001;60(4):1240–8. 10.1046/j.1523-1755.2001.00963.x . 11576338

[11] Shillingford JM, Murcia NS, Larson CH, Low SH, Hedgepeth R, Brown N, et al The mTOR pathway is regulated by polycystin-1, and its inhibition reverses renal cystogenesis in polycystic kidney disease. Proceedings of the National Academy of Sciences. 2006;103(14):5466–71. 10.1073/pnas.0509694103 16567633 PMC1459378

[12] Sweeney WE, Frost P, Avner ED. Tesevatinib ameliorates progression of polycystic kidney disease in rodent models of autosomal recessive polycystic kidney disease. World J Nephrol. 2017;6(4):188–200. Epub 2017/07/22. 10.5527/wjn.v6.i4.188 ; PubMed Central PMCID: PMCPMC5500456. 28729967 PMC5500456

[13] Lian P, Li A, Li Y, Liu H, Liang D, Hu B, et al Loss of Polycystin-1 Inhibits Bicc1 Expression during Mouse Development. PLoS One. 2014;9(3):e88816 Epub 2014/03/07. 10.1371/journal.pone.0088816 ; PubMed Central PMCID: PMCPMC3940423. 24594709 PMC3940423

[14] Tran U, Zakin L, Schweickert A, Agrawal R, Döger R, Blum M, et al The RNA-binding protein bicaudal C regulates polycystin 2 in the kidney by antagonizing miR-17 activity. Development. 2010;137(7):1107–16. 10.1242/dev.046045 20215348 PMC2835326

[15] Kraus MR, Clauin S, Pfister Y, Di Maio M, Ulinski T, Constam D, et al Two mutations in human BICC1 resulting in Wnt pathway hyperactivity associated with cystic renal dysplasia. Human mutation. 2012;33(1):86–90. 10.1002/humu.21610 . 21922595

[16] Piazzon N, Maisonneuve C, Guilleret I, Rotman S, Constam DB. Bicc1 links the regulation of cAMP signaling in polycystic kidneys to microRNA-induced gene silencing. Journal of molecular cell biology. 2012;4(6):398–408. 10.1093/jmcb/mjs027 . 22641646

[17] Piazzon N, Bernet F, Guihard L, Leonhard WN, Urfer S, Firsov D, et al Urine Fetuin-A is a biomarker of autosomal dominant polycystic kidney disease progression. Journal of translational medicine. 2015;13:103 10.1186/s12967-015-0463-7 ; PubMed Central PMCID: PMC4416261. 25888842 PMC4416261

[18] Knight MJ, Leettola C, Gingery M, Li H, Bowie JU. A human sterile alpha motif domain polymerizome. Protein Sci. 2011;20(10):1697–706. 10.1002/pro.703 ; PubMed Central PMCID: PMC3218363. 21805519 PMC3218363

[19] Rothé B, Leal-Esteban L, Bernet F, Urfer S, Doerr N, Weimbs T, et al Bicc1 Polymerization Regulates the Localization and Silencing of Bound mRNA. Molecular and Cellular Biology. 2015;35(19):3339–53. 10.1128/MCB.00341-15 26217012 PMC4561730

[20] Maisonneuve C, Guilleret I, Vick P, Weber T, Andre P, Beyer T, et al Bicaudal C, a novel regulator of Dvl signaling abutting RNA-processing bodies, controls cilia orientation and leftward flow. Development. 2009;136(17):3019–30. 10.1242/dev.038174 . 19666828

[21] Park S, Blaser S, Marchal MA, Houston DW, Sheets MD. A gradient of maternal Bicaudal-C controls vertebrate embryogenesis via translational repression of mRNAs encoding cell fate regulators. Development. 2016;143(5):864–71. 10.1242/dev.131359 26811381 PMC4813341

[22] Gamberi C, Hipfner DR, Trudel M, Lubell WD. Bicaudal C mutation causes myc and TOR pathway up-regulation and polycystic kidney disease-like phenotypes in Drosophila. PLoS genetics. 2017;13(4):e1006694 10.1371/journal.pgen.1006694 ; PubMed Central PMCID: PMC5390980. 28406902 PMC5390980

[23] Zhang Y, Cooke A, Park S, Dewey CN, Wickens M, Sheets MD. Bicaudal-C spatially controls translation of vertebrate maternal mRNAs. RNA. 2013;9(11):1575–82. 10.1261/rna.041665.113 . 24062572 PMC3851724

[24] Chicoine J, Benoit P, Gamberi C, Paliouras M, Simonelig M, Lasko P. Bicaudal-C recruits CCR4-NOT deadenylase to target mRNAs and regulates oogenesis, cytoskeletal organization, and its own expression. Developmental cell. 2007;13(5):691–704. 10.1016/j.devcel.2007.10.002 . 17981137

[25] Iaconis D, Monti M, Renda M, van Koppen A, Tammaro R, Chiaravalli M, et al The centrosomal OFD1 protein interacts with the translation machinery and regulates the synthesis of specific targets. Sci Rep. 2017;7(1):1224 Epub 2017/04/30. 10.1038/s41598-017-01156-x . 28450740 PMC5430665

[26] Castagnetti S, Ephrussi A. Orb and a long poly(A) tail are required for efficient oskar translation at the posterior pole of the Drosophila oocyte. Development. 2003;130(5):835–43. Epub 2003/01/23. . 12538512 10.1242/dev.00309

[27] Kugler JM, Chicoine J, Lasko P. Bicaudal-C associates with a Trailer Hitch/Me31B complex and is required for efficient Gurken secretion. Developmental biology. 2009;328(1):160–72. 10.1016/j.ydbio.2009.01.024 ; PubMed Central PMCID: PMC2684517. 19389362 PMC2684517

[28] Chen T, Damaj BB, Herrera C, Lasko P, Richard S. Self-association of the single-KH-domain family members Sam68, GRP33, GLD-1, and Qk1: role of the KH domain. Mol Cell Biol. 1997;17(10):5707–18. 9315629 10.1128/mcb.17.10.5707 PMC232419

[29] Stagner EE, Bouvrette DJ, Cheng J, Bryda EC. The polycystic kidney disease-related proteins Bicc1 and SamCystin interact. Biochemical and biophysical research communications. 2009;383(1):16–21. 10.1016/j.bbrc.2009.03.113 . 19324013

[30] Hoff S, Halbritter J, Epting D, Frank V, Nguyen T-MT, van Reeuwijk J, et al ANKS6 is a central component of a nephronophthisis module linking NEK8 to INVS and NPHP3. Nature genetics. 2013;45(8):951–6. 10.1038/ng.2681 23793029 PMC3786259

[31] Yakulov TA, Yasunaga T, Ramachandran H, Engel C, Muller B, Hoff S, et al Anks3 interacts with nephronophthisis proteins and is required for normal renal development. Kidney international. 2015;87(6):1191–200. 10.1038/ki.2015.17 . 25671767

[32] Mohieldin AM, Haymour HS, Lo ST, AbouAlaiwi WA, Atkinson KF, Ward CJ, et al Protein composition and movements of membrane swellings associated with primary cilia. Cellular and molecular life sciences: CMLS. 2015;72(12):2415–29. 10.1007/s00018-015-1838-x ; PubMed Central PMCID: PMC4503369. 25650235 PMC4503369

[33] Ramachandran H, Engel C, Muller B, Dengjel J, Walz G, Yakulov TA. Anks3 alters the sub-cellular localization of the Nek7 kinase. Biochem Biophys Res Commun. 2015 10.1016/j.bbrc.2015.07.063 . 26188091

[34] Kobayashi N, Yang J, Ueda A, Suzuki T, Tomaru K, Takeno M, et al RanBPM, Muskelin, p48EMLP, p44CTLH, and the armadillo-repeat proteins ARMC8alpha and ARMC8beta are components of the CTLH complex. Gene. 2007;396(2):236–47. 10.1016/j.gene.2007.02.032 . 17467196

[35] Francis O, Han F, Adams JC. Molecular phylogeny of a RING E3 ubiquitin ligase, conserved in eukaryotic cells and dominated by homologous components, the muskelin/RanBPM/CTLH complex. PLoS One. 2013;8(10):e75217 10.1371/journal.pone.0075217 ; PubMed Central PMCID: PMC3797097. 24143168 PMC3797097

[36] Hammerle M, Bauer J, Rose M, Szallies A, Thumm M, Dusterhus S, et al Proteins of newly isolated mutants and the amino-terminal proline are essential for ubiquitin-proteasome-catalyzed catabolite degradation of fructose-1,6-bisphosphatase of Saccharomyces cerevisiae. J Biol Chem. 1998;273(39):25000–5. Epub 1998/09/17. . 9737955 10.1074/jbc.273.39.25000

[37] Santt O, Pfirrmann T, Braun B, Juretschke J, Kimmig P, Scheel H, et al The yeast GID complex, a novel ubiquitin ligase (E3) involved in the regulation of carbohydrate metabolism. Mol Biol Cell. 2008;19(8):3323–33. 10.1091/mbc.E08-03-0328 ; PubMed Central PMCID: PMC2488282. 18508925 PMC2488282

[38] Chen SJ, Wu X, Wadas B, Oh JH, Varshavsky A. An N-end rule pathway that recognizes proline and destroys gluconeogenic enzymes. Science. 2017;355(6323). 10.1126/science.aal3655 . 28126757 PMC5457285

[39] Regelmann J. Catabolite Degradation of Fructose-1,6-bisphosphatase in the Yeast Saccharomyces cerevisiae: A Genome-wide Screen Identifies Eight Novel GID Genes and Indicates the Existence of Two Degradation Pathways. Molecular Biology of the Cell. 2002;14(4):1652–63. 10.1091/mbc.E02-08-0456 12686616 PMC153129

[40] Fu Y, Kim I, Lian P, Li A, Zhou L, Li C, et al Loss of Bicc1 impairs tubulomorphogenesis of cultured IMCD cells by disrupting E-cadherin-based cell-cell adhesion. Eur J Cell Biol. 2010;89(6):428–36. 10.1016/j.ejcb.2010.01.002 ; PubMed Central PMCID: PMC2886128. 20219263 PMC2886128

[41] Rothe B, Leettola CN, Leal-Esteban L, Cascio D, Fortier S, Isenschmid M, et al Crystal Structure of Bicc1 SAM Polymer and Mapping of Interactions between the Ciliopathy-Associated Proteins Bicc1, ANKS3, and ANKS6. Structure. 2018;26(2):209–24 e6. 10.1016/j.str.2017.12.002 . 29290488 PMC6258031

[42] Menssen R, Schweiggert Jr, Schreiner J, KuÅ¡eviÄ‡ D, Reuther J, Braun B, et al Exploring the Topology of the Gid Complex, the E3 Ubiquitin Ligase Involved in Catabolite-induced Degradation of Gluconeogenic Enzymes. The Journal of Biological Chemistry. 2012;287(30):25602–14. 10.1074/jbc.M112.363762 PMC3408164. 22645139 PMC3408164

[43] Dohner K, Ramos-Nascimento A, Bialy D, Anderson F, Hickford-Martinez A, Rother F, et al Importin alpha1 is required for nuclear import of herpes simplex virus proteins and capsid assembly in fibroblasts and neurons. PLoS pathogens. 2018;14(1):e1006823 10.1371/journal.ppat.1006823 ; PubMed Central PMCID: PMC5773220. 29304174 PMC5773220

[44] Schlimpert M, Lagies S, Budnyk V, Muller B, Walz G, Kammerer B. Metabolic Phenotyping of Anks3 Depletion in mIMCD-3 cells—a Putative Nephronophthisis Candidate. Scientific reports. 2018;8(1):9022 10.1038/s41598-018-27389-y . 29899363 PMC5998149

[45] Huang M, Graves LM. De novo synthesis of pyrimidine nucleotides; emerging interfaces with signal transduction pathways. Cell Mol Life Sci. 2003;60(2):321–36. Epub 2003/04/08. . 12678497 10.1007/s000180300027 PMC11146060

[46] Mashima T, Seimiya H, Tsuruo T. De novo fatty-acid synthesis and related pathways as molecular targets for cancer therapy. Br J Cancer. 2009;100(9):1369–72. 10.1038/sj.bjc.6605007 ; PubMed Central PMCID: PMC2694429. 19352381 PMC2694429

[47] Menssen R, Schweiggert Jr, Schreiner J, KuÅ¡eviÄ‡ D, Reuther J, Braun B, et al Exploring the Topology of the Gid Complex, the E3 Ubiquitin Ligase Involved in Catabolite-induced Degradation of Gluconeogenic Enzymes. The Journal of Biological Chemistry. 2012;287(30):25602–14. 10.1074/jbc.M112.363762 PMC3408164. 22645139 PMC3408164

[48] Pitre S, Dehne F, Chan A, Cheetham J, Duong A, Emili A, et al PIPE: a protein-protein interaction prediction engine based on the re-occurring short polypeptide sequences between known interacting protein pairs. BMC Bioinformatics. 2006;7:365 Epub 2006/07/29. 10.1186/1471-2105-7-365 ; PubMed Central PMCID: PMCPMC1557541. 16872538 PMC1557541

[49] Zhu Y, Wang Y, Xia C, Li D, Li Y, Zeng W, et al WDR26: a novel Gbeta-like protein, suppresses MAPK signaling pathway. J Cell Biochem. 2004;93(3):579–87. 10.1002/jcb.20175 . 15378603

[50] Higa LA, Wu M, Ye T, Kobayashi R, Sun H, Zhang H. CUL4-DDB1 ubiquitin ligase interacts with multiple WD40-repeat proteins and regulates histone methylation. Nat Cell Biol. 2006;8(11):1277–83. Epub 2006/10/17. 10.1038/ncb1490 . 17041588

[51] Nakamura M, Masuda H, Horii J, Kuma K, Yokoyama N, Ohba T, et al When overexpressed, a novel centrosomal protein, RanBPM, causes ectopic microtubule nucleation similar to gamma-tubulin. J Cell Biol. 1998;143(4):1041–52. Epub 1998/11/17. ; PubMed Central PMCID: PMCPMC2132962. 9817760 10.1083/jcb.143.4.1041 PMC2132962

[52] Dishinger JF, Kee HL, Jenkins PM, Fan S, Hurd TW, Hammond JW, et al Ciliary entry of the kinesin-2 motor KIF17 is regulated by importin-beta2 and RanGTP. Nat Cell Biol. 2010;12(7):703–10. Epub 2010/06/08. doi: ncb2073 [pii] 10.1038/ncb2073 ; PubMed Central PMCID: PMC2896429. 20526328 PMC2896429

[53] Fan S, Margolis B. The Ran importin system in cilia trafficking. Organogenesis. 2011;7(3):147–53. Epub 2011/07/28. 10.4161/org.7.3.17084 ; PubMed Central PMCID: PMCPMC3243027. 21791971 PMC3243027

[54] Menon RP, Gibson TJ, Pastore A. The C terminus of fragile X mental retardation protein interacts with the multi-domain Ran-binding protein in the microtubule-organising centre. J Mol Biol. 2004;343(1):43–53. Epub 2004/09/24. 10.1016/j.jmb.2004.08.024 . 15381419

[55] Zhao Y, Peng S, Jia C, Xu F, Xu Y, Dai C. Armc8 regulates the invasive ability of hepatocellular carcinoma through E-cadherin/catenin complex. Tumour Biol. 2016;37(8):11219–24. Epub 2016/03/06. 10.1007/s13277-016-5006-1 . 26944057

[56] Jiang G, Zhang Y, Zhang X, Fan C, Wang L, Xu H, et al ARMc8 indicates aggressive colon cancers and promotes invasiveness and migration of colon cancer cells. Tumour Biol. 2015;36(11):9005–13. Epub 2015/06/18. 10.1007/s13277-015-3664-z . 26081621

[57] Jiang G, Yang D, Wang L, Zhang X, Xu H, Miao Y, et al A novel biomarker ARMc8 promotes the malignant progression of ovarian cancer. Hum Pathol. 2015;46(10):1471–9. Epub 2015/08/04. 10.1016/j.humpath.2015.06.004 . 26232863

[58] Suzuki T, Ueda A, Kobayashi N, Yang J, Tomaru K, Yamamoto M, et al Proteasome-dependent degradation of alpha-catenin is regulated by interaction with ARMc8alpha. Biochem J. 2008;411(3):581–91. 10.1042/BJ20071312 . 18215130

[59] Gerich JE. Role of the kidney in normal glucose homeostasis and in the hyperglycaemia of diabetes mellitus: therapeutic implications. Diabet Med. 2010;27(2):136–42. 10.1111/j.1464-5491.2009.02894.x ; PubMed Central PMCID: PMC4232006. 20546255 PMC4232006

[60] Curthoys NP, Moe OW. Proximal tubule function and response to acidosis. Clin J Am Soc Nephrol. 2014;9(9):1627–38. Epub 2013/08/03. 10.2215/CJN.10391012 ; PubMed Central PMCID: PMCPMC4152816. 23908456 PMC4152816

[61] Preuss H, Geoly K, Johnson M, Chester A, Kliger A, Schreiner G. Tubular function in adult polycystic kidney disease. Nephron. 1979;24(4):198–204. Epub 1979/01/01. 10.1159/000181715 . 40149

[62] Tolins JP, Hostetter MK, Hostetter TH. Hypokalemic nephropathy in the rat. Role of ammonia in chronic tubular injury. J Clin Invest. 1987;79(5):1447–58. Epub 1987/05/01. 10.1172/JCI112973 ; PubMed Central PMCID: PMCPMC424417. 3553240 PMC424417

[63] Torres VE, Mujwid DK, Wilson DM, Holley KH. Renal cystic disease and ammoniagenesis in Han:SPRD rats. J Am Soc Nephrol. 1994;5(5):1193–200. Epub 1994/11/01. . 7873729 10.1681/ASN.V551193

[64] Chen S-J, Wu X, Wadas B, Oh J-H, Varshavsky A. An N-end rule pathway that recognizes proline and destroys gluconeogenic enzymes. Science. 2017;355(6323). 10.1126/science.aal3655 28126757 PMC5457285

[65] Dong C, Yuan T, Wu Y, Wang Y, Fan TW, Miriyala S, et al Loss of FBP1 by Snail-mediated repression provides metabolic advantages in basal-like breast cancer. Cancer Cell. 2013;23(3):316–31. Epub 2013/03/05. 10.1016/j.ccr.2013.01.022 ; PubMed Central PMCID: PMCPMC3703516. 23453623 PMC3703516

[66] Li B, Qiu B, Lee DS, Walton ZE, Ochocki JD, Mathew LK, et al Fructose-1,6-bisphosphatase opposes renal carcinoma progression. Nature. 2014;513(7517):251–5. 10.1038/nature13557 ; PubMed Central PMCID: PMC4162811. 25043030 PMC4162811

[67] Ran FA, Hsu PD, Lin CY, Gootenberg JS, Konermann S, Trevino AE, et al Double nicking by RNA-guided CRISPR Cas9 for enhanced genome editing specificity. Cell. 2013;154(6):1380–9. Epub 2013/09/03. 10.1016/j.cell.2013.08.021 . 23992846 PMC3856256

[68] Garcia-Bermudez J, Sanchez-Arago M, Soldevilla B, Del Arco A, Nuevo-Tapioles C, Cuezva JM. PKA Phosphorylates the ATPase Inhibitory Factor 1 and Inactivates Its Capacity to Bind and Inhibit the Mitochondrial H(+)-ATP Synthase. Cell Rep. 2015;12(12):2143–55. 10.1016/j.celrep.2015.08.052 . 26387949

[69] Gloeckner CJ, Boldt K, Schumacher A, Ueffing M. Tandem affinity purification of protein complexes from mammalian cells by the Strep/FLAG (SF)-TAP tag. Methods in molecular biology. 2009;564:359–72. 10.1007/978-1-60761-157-8_21 . 19544034

[70] Chopra T, Hamelin R, Armand F, Chiappe D, Moniatte M, McKinney JD. Quantitative mass spectrometry reveals plasticity of metabolic networks in Mycobacterium smegmatis. Mol Cell Proteomics. 2014;13(11):3014–28. Epub 2014/07/07. 10.1074/mcp.M113.034082 ; PubMed Central PMCID: PMCPMC4223488. 24997995 PMC4223488

[71] Gietz RD, Schiestl RH. High-efficiency yeast transformation using the LiAc/SS carrier DNA/PEG method. Nat Protoc. 2007;2(1):31–4. 10.1038/nprot.2007.13 . 17401334

