# Supplementary figures and images for: Role of Bicaudal C1 in renal gluconeogenesis and its novel interaction with the CTLH complex
Source: PLoS Genet. 2018 Jul 11;14(7):e1007487. doi: 10.1371/journal.pgen.1007487 (PMC6056059; doi:10.1371/journal.pgen.1007487)

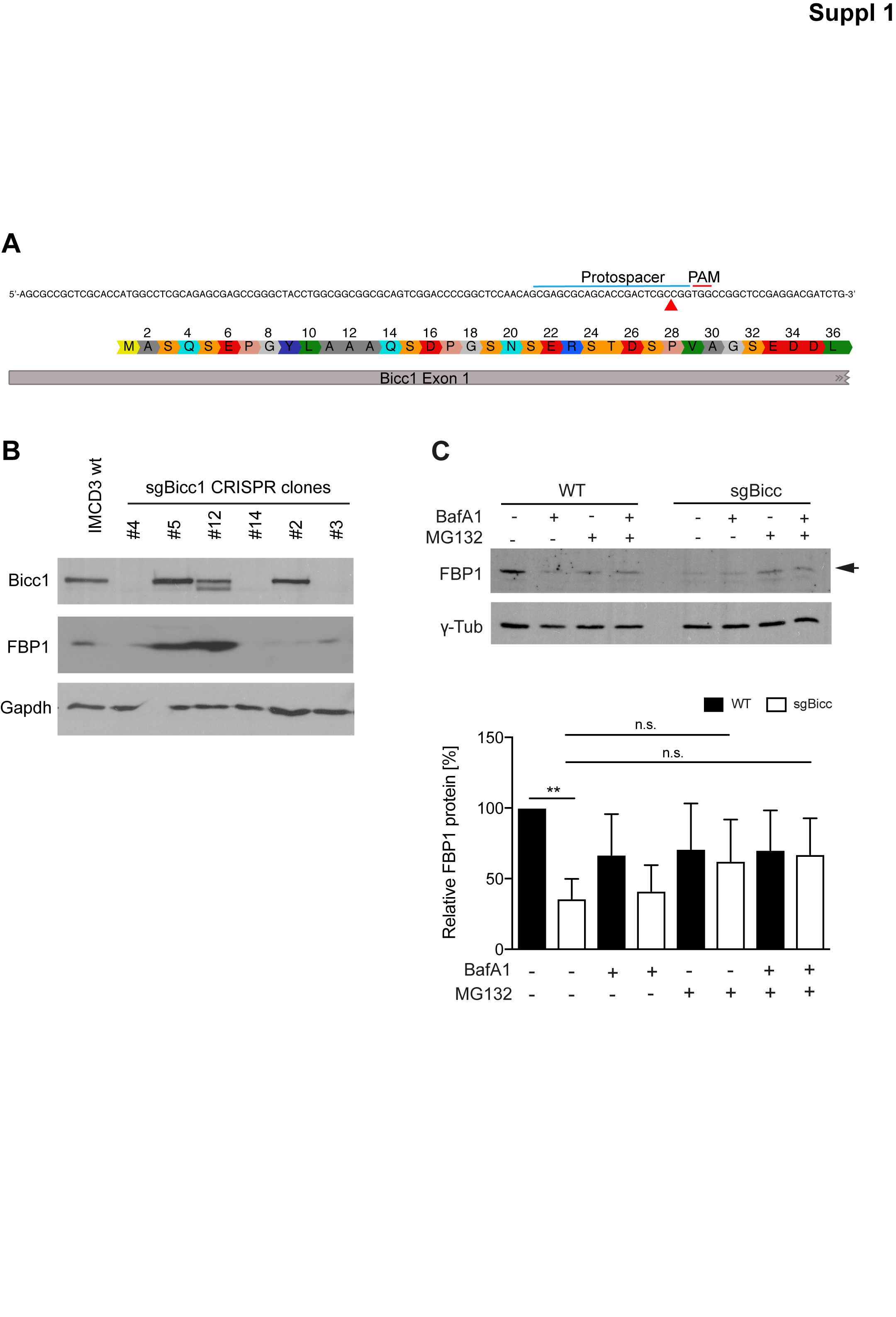

Supplement: S1 Fig — (A) Schematic of Bicc1 targeting. (B) Bicc1 and FBP1 Western blot of CRISPR/Cas9 treated mIMCD3 cell clones. (C) FBP1 protein levels in mIMCD3 cells after treatment with Bafilomycin A1 (BafA1, 100 nM), MG132 [10 μM] or both for 4 hrs. Bars represent mean ± SEM fold changes in Bicc1 protein levels relative to untreated cells in two experiments (**p <0.01). (TIF) [file pgen.1007487.s001.tif]

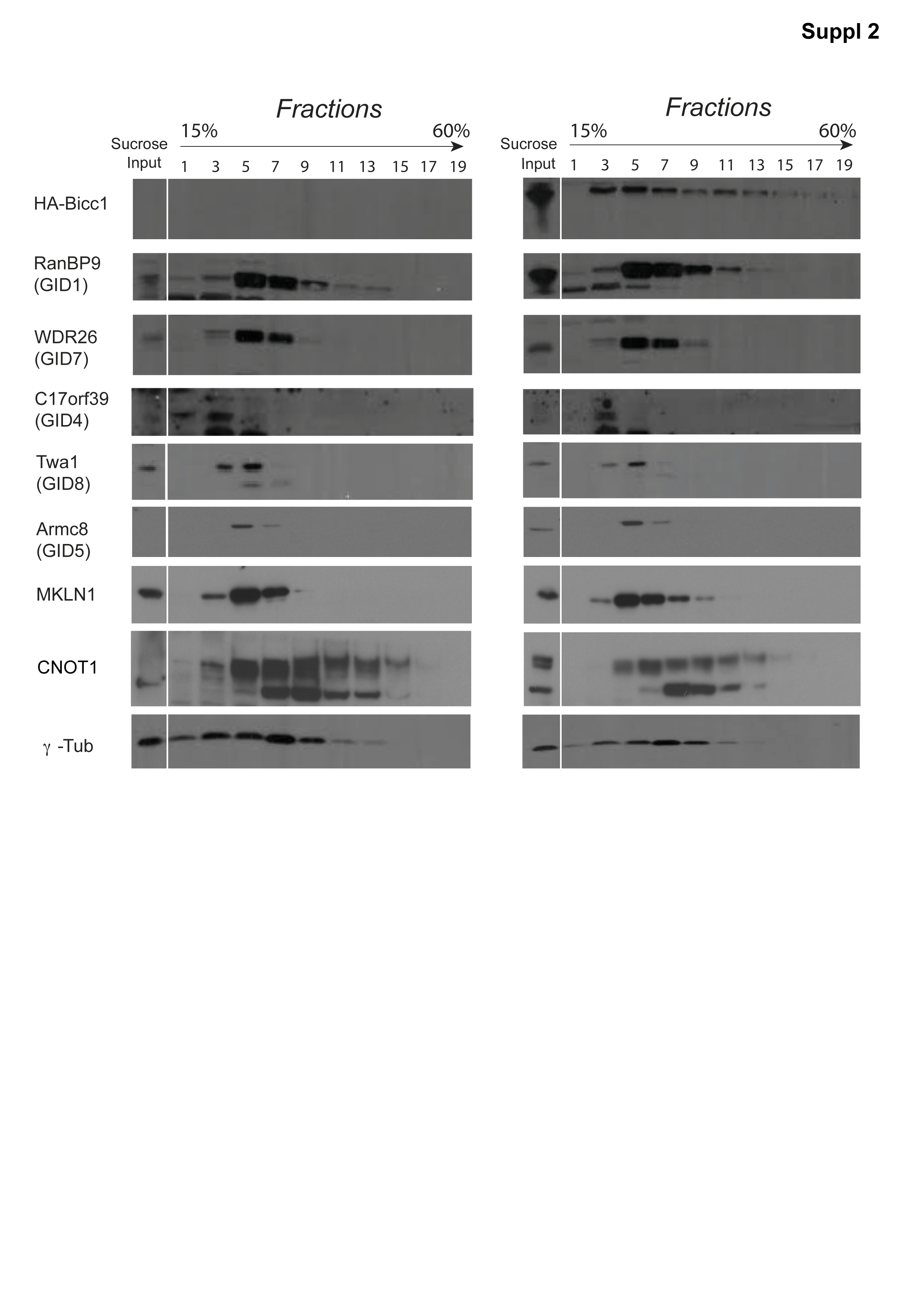

Supplement: S2 Fig — Extracts of HEK293T transfected with HA-Bicc1 or empty vector (mock) were fractionated on a continuous 15 to 60% sucrose gradient. Arrows indicate the order for collecting the fractions (top to bottom). Inputs and fractionated samples were on the same gel but shown at different exposure times. (TIF) [file pgen.1007487.s002.tif]

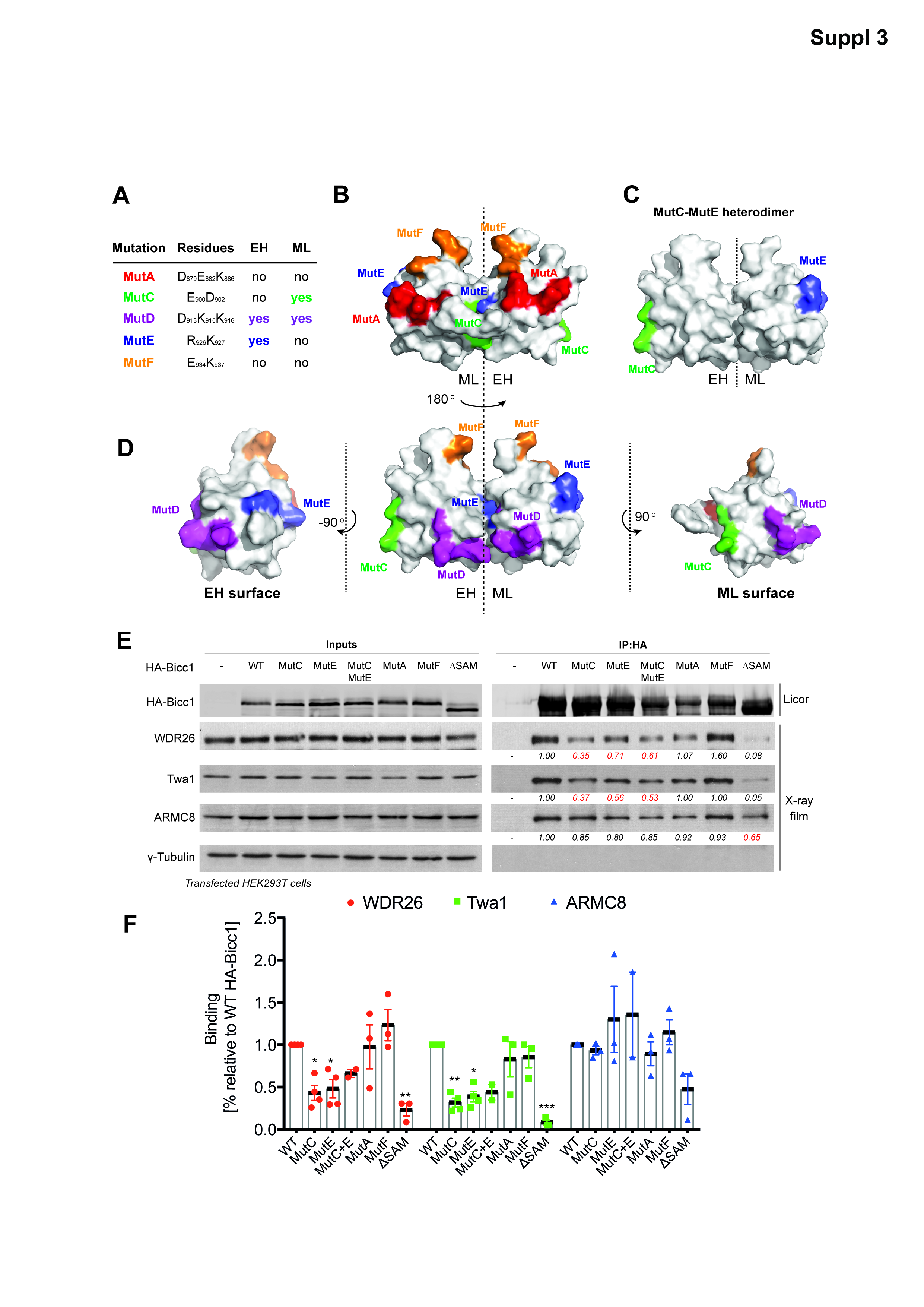

Supplement: S3 Fig — (A) Positions of mutations in the Bicc1 SAM domain, and their effects on the integrity of EH or ML surfaces [19]. (B) Space filling model of a Bicc1 SAM domain dimer from an angle viewing the positions of the control mutations MutA and MutF in the first or fifth α-helix, respectively, outside the ML and EH surfaces of the SAM-SAM interface. (C) Backside view of a SAM domain heterodimer of co-expressed Bicc1 MutC and Bicc1 MutE associating through their wild-type EH and ML surfaces, respectively, so that MutC or MutE mutations at the extremities prevent polymer extension. (D) As in (C), but with individual SAM subunits rotated along their vertical axis to display frontal views of their EH (left) or ML surface (right). The position of the mutation MutD (purple) encompasses both surfaces. (E) Western blot analysis of the indicated CTLH complex subunits after co-immunoprecipitation with HA-Bicc1 or polymerization mutant derivatives. γ-tubulin was a loading control. Inputs represent 2% of cell extracts. Numbers below each panel indicate the ratio of protein that coprecipitated with the indicated polymerization mutant HA-Bicc1, divided by the amount pulled down by wild-type control. HA-Bicc1 was imaged by a LI-COR Odyssey CLx system to avoid signal saturation. (F) Mean values ± SEM from 4 independent experiments are shown below. P values were estimated using 2-way Anova and Dunnet's multiple comparison test (*p<0.05, **p <0.01, ***p <0.001). (TIF) [file pgen.1007487.s003.tif]

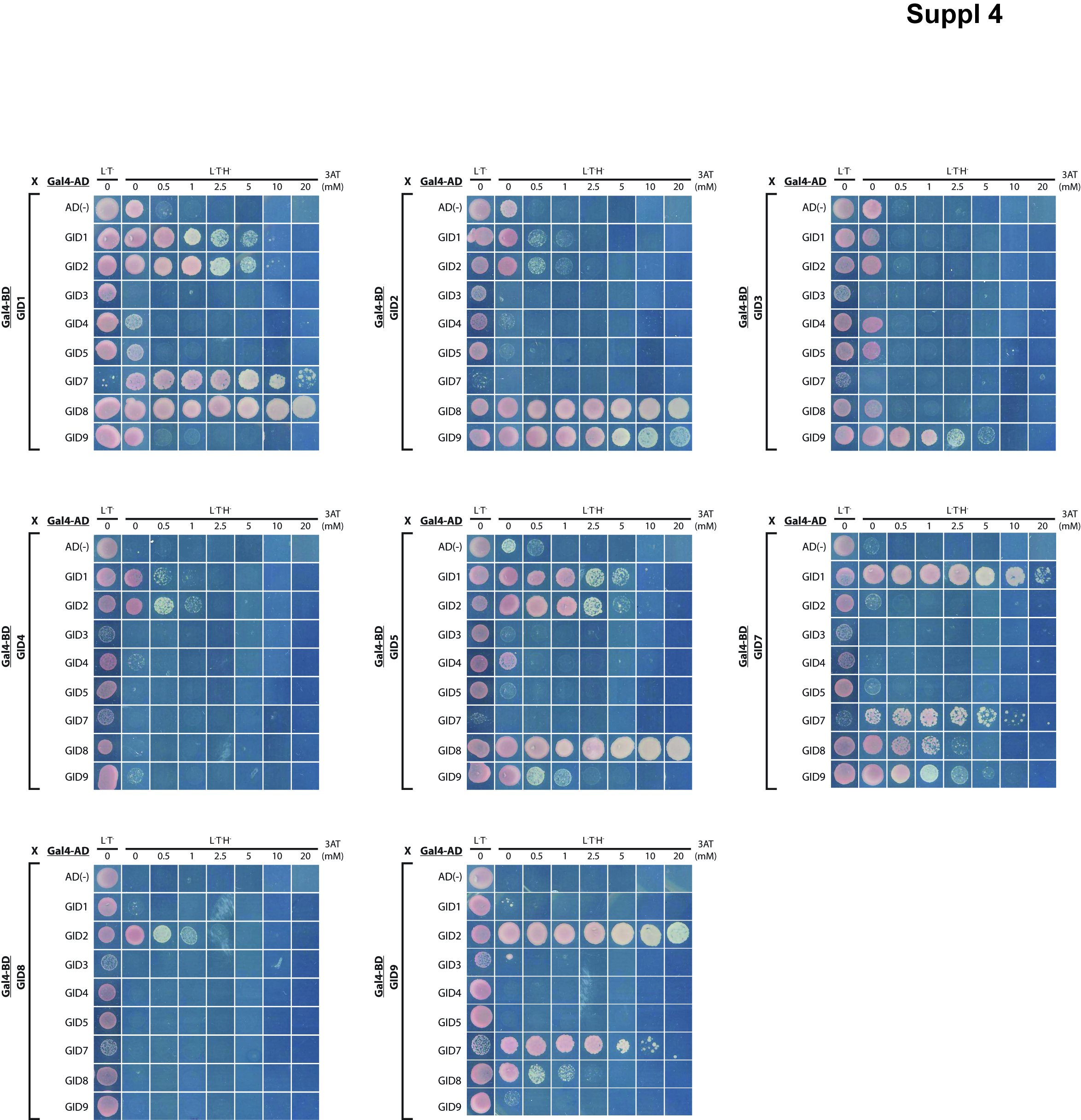

Supplement: S4 Fig — Pairs of CTLH subunits fused to Gal4-AD bait or Gal4-DBD prey proteins induce cell growth if they bind each other. Each CTLH subunit was tested both as bait and prey. Empty Gal4-AD was a negative control. Titration of the competitive HIS3 antagonist 3‐Amino‐1,2,4‐triazol (3AT) served to assess the strength of each interaction. Data are representative of 2 experiments with similar results. (TIF) [file pgen.1007487.s004.tif]

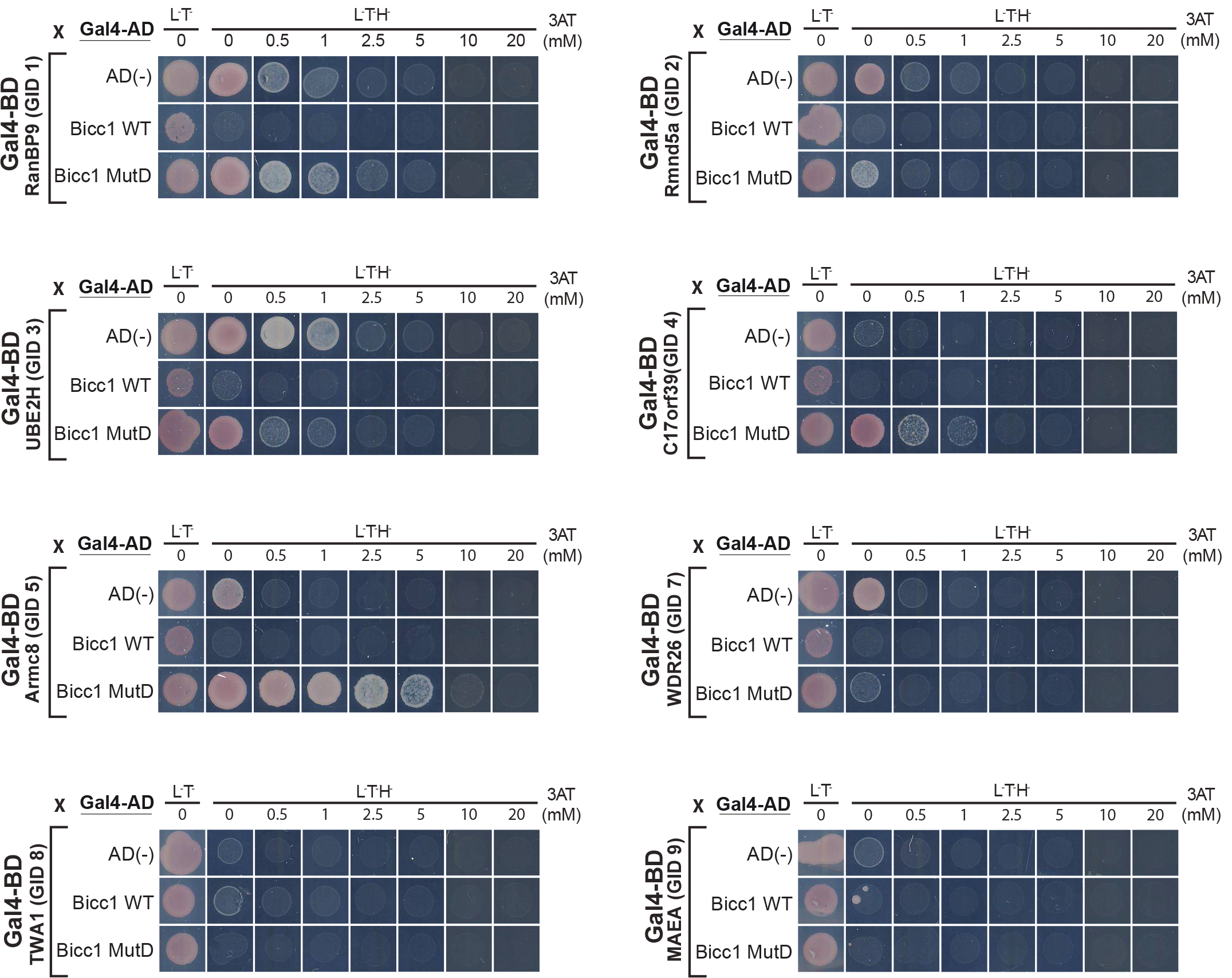

Supplement: S5 Fig — Yeast two-hybrid assays of the indicated bait and prey fusion proteins. Data are representative of 2 experiments with similar results. (TIF) [file pgen.1007487.s005.tif]

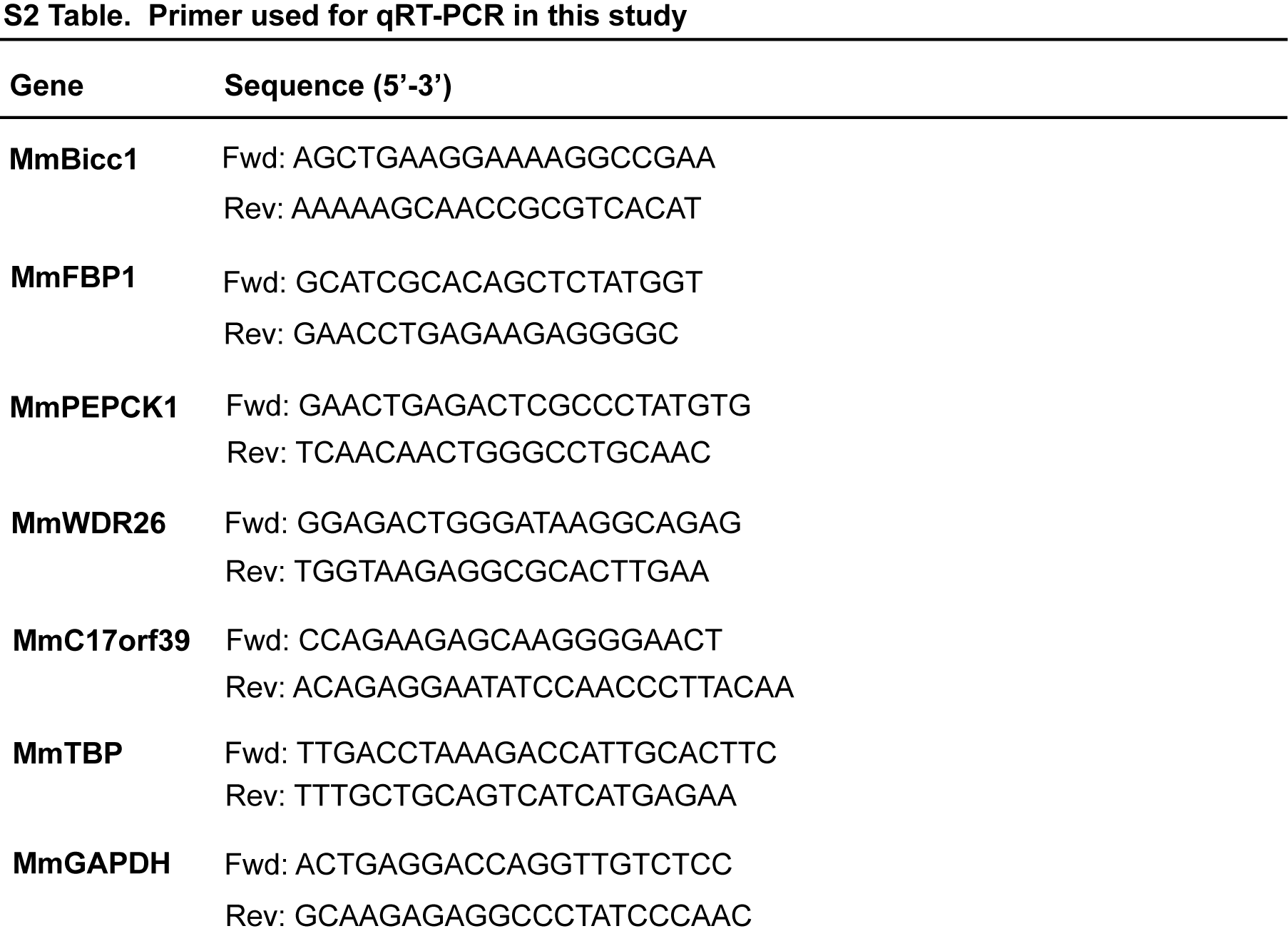

Supplement: S2 Table — (TIF) [file pgen.1007487.s007.tif]
